# Supplementary material for: Anti-Fouling Properties of Phosphonium Ionic Liquid Coatings in the Marine Environment
Source: Polymers (Basel). 2023 Sep 6;15(18):3677. doi: 10.3390/polym15183677 (PMC10534580; doi:10.3390/polym15183677)
Supplement: Supplementary file 1 [file polymers-15-03677-s001.zip › polymers-2521897-supplementary.pdf]

polymers

MDPI

Supplementary Materials
Anti-fouling Properties of Phosphonium Ionic Liquid Coatings in the Marine Environment

Sajith Kaniyadan Baiju [1,2,3], Brent James Martin [4], Rayleen Fredericks [1,2,3], Harikrishnan Raghavan [1,3], Karnika De Silva [5] and Matthew Greig Cowan [1,2,3,*]

[1] Department of Chemical and Process Engineering, University of Canterbury, Private Bag 4800, Christchurch 8140, New Zealand; sajith.kaniyadanbaiju@pg.canterbury.ac.nz (S.K.B.); rayleen.fredericks@canterbury.ac.nz (R.F.)
[2] New Zealand Product Accelerator, University of Canterbury, Private Bag 4800, Christchurch 8140, New Zealand
[3] MacDiarmid Institute for Advanced Materials and Nanotechnology, University of Canterbury, Private Bag 4800, Christchurch 8140, New Zealand
[4] Defence Technology Agency (DTA), Private Bag 32901, Auckland 0744, New Zealand
[5] NZ Product Accelerator, Faculty of Engineering, University of Auckland, Auckland 1010, New Zealand
* Correspondence: matthew.cowan@canterbury.ac.nz; Tel.: +64-3369-2521

Contents

Contents 1

1. Summaries of literature searches 3

2. Ionic Liquid Characterization Data 4

2.1. Tributyl(vinylbenzyl)phosphonium Chloride ([P_{444VB}][Cl]) 4

2.1.1. 1H NMR (**Figure S1**) 4

2.1.2. MS (**Figure S2**) 5

2.2. Tributyl(octyl)phosphonium Bromide ([P_{4448}][Br]) 5

2.2.1. ^{1}H NMR (**Figure S3**) 5

2.2.2. MS (**Figure S4**) 6

2.3. Tributyl(vinylbenzyl)phosphonium Docusate ([P_{444VB}] [AOT]) 6

2.3.1. ^{1}H NMR (**Figure S5**) 6

2.3.2. FT-IR (**Figure S6**) 7

2.3.3. MS (**Figure S7**, **Figure S8**) 8

2.4. Tributyl(octyl)phosphonium Docusate ([P_{4448}][AOT]) 9

2.4.1. ^{1}H NMR (**Figure S9**) 9

2.4.2. FT-IR (**Figure S10**) 10

2.4.3. MS (**Figure S11**, **Figure S12**) 11

2.5. Trioctyl(vinylbenzyl)phosphonium Chloride ([P_{888VB}][Cl]) 11

2.5.1. ^{1}H NMR (**Figure S13**) 11

2.5.2. FT-IR (**Figure S14**)........ 12

2.6. Trioctyl(tetradecyl)phosphonium Bromide ([P_{88814}][Br]) 14

2.6.1. ^{1}H NMR (**Figure S16**) 14

2.6.2. FT-IR (**Figure S17**)........ 14

2.6.3. MS (**Figure S18**)........ 15

2.7. Trioctyl(vinylbenzyl)phosphonium Docusate ([P_{888VB}] [AOT]) 16

2.7.1. ^{1}H NMR (**Figure S19**) 16

2.7.2. ^{13}C NMR (**Figure S20**)........ 16

2.7.3. COSY (**Figure S21**) 17

2.7.4. FT-IR (**Figure S22**)........ 18

2.7.5. MS (**Figure S23**, **Figure S24**)........ 19

2.8. Trioctyl(tetradecyl)phosphonium Docusate ([P_{88814}] [AOT])........ 19

2.8.1. ^{1}H NMR (**Figure S25**) 19

2.8.2. ^{13}C NMR (**Figure S26**)........ 20

2.8.3. COSY (**Figure S27**) 21

2.8.4. FT-IR (**Figure S28**)........ 21

2.8.5. MS (**Figure S29**, **Figure S30**)........ 22

3. Characterization of Polymerization 23

4. Contact Angle Measurement........24

5. Micro Testing........26

5.1. Fluorescence Assay 26

5.2. Disk Diffusion Assay 27

6. Total fouling rate calculation........29

6.1. Field Test Picture (Tributyl(vinylbenzyl)phosphonium docusate 30

7. Surface Roughness of the Gel Samples 33

8. Reference........34

1. Summaries of literature searches

The search was performed over a period of 26/5/2022-7/12/2022 using the 'Article Search' function of Google Scholar, the University of Canterbury Library Database, and Scopus.

The following key phrase was used:

"Antifouling properties of Ionic Liquids", "Antifouling properties of phosphonium-based Ionic Liquid", "Antifouling properties of imidazolium-based ionic liquids", "Antifouling properties of Pyridium-based ionic Liquid".

The results were filtered by looking at the following terms in the title and abstract: "antifouling", "ionic liquids", and "anti-fouling". Duplicates were removed and the list was compiled, resulting in 74 references (Table S1). The results were judged by reading the abstract and data from the relevant papers were recorded.

Table S1: Summary of literature review (1) results before filtering.

Copyright: © 2023 by the authors. Licensee MDPI, Basel, Switzerland. This article is an open access article distributed under the terms and conditions of the Creative Commons Attribution (CC BY) license (https://creativecommons.org/licenses/by/4.0/).

**Topic**	**Database**	**Number of Results before filtering**	**Filtering Criteria**	**Number of Results after Filtering**
Antifouling properties of Ionic Liquids	Google Scholar	28600	A quick read of the title and the abstract of the first 60 papers for the selective keywords	16
	Scopus	76		20
	UC Library Search	114		13
Antifouling properties of phosphonium-based ionic Liquid	Google Scholar	2500		6
	Scopus	0		
	UC Library Search	0		
Antifouling properties of imidazolium-based ionic liquids	Google Scholar	2820		40
	Scopus	2		
	UC Library Search	7		
Antifouling properties of Pyridium-based ionic Liquid	Google Scholar	83		0
	Scopus	0		
	UC Library Search	0		

Another search was performed on 16/01/2023 at 13:43 PM using the article search function of SciFinder^{n}.

The following key phrase was used: "Antifouling properties of Ionic Liquid" which resulted in a total number of 148,659 references.

The results were filtered by the criteria shown in Table S2:

Table S2: Summary of literature Review (2) result before filtering

Language	English
Document Type	Journal, Patent
Concept	Antifouling agents, Antifouling coating materials.
Formulation Purpose	Antifouling agents, antibacterial agents, antifouling coating materials, antimicrobial agents, biofouling control agents, antibiofilm agents, biocides, antibacterial coating materials, antifouling paints, marine antifouling agents, marine antifouling coating materials, marine coating materials, UV- curable coating materials.

71 references were gathered as a result. Reviewing the abstracts allowed for appraisal, and pertinent data from the studies was recorded. 25 additional publications were identified from this literature search.

2. Ionic Liquid Characterization Data

All ionic liquids were characterized by their ^{1}H NMR spectra and +ve ion mass spectrometry data to verify the correct proton structure and molecular weight of the cation species. Ionic liquids exchanged to the docusate anion were characterized through infrared spectroscopy (IR) to verify the presence of the sulfonate asymmetric stretch (ca. 1350 cm^{-1}) and symmetric stretches (ca. 1175 cm^{-1}) and carbonyl stretches (ca. 1750 cm^{-1}) through comparison to the IR spectrum of sodium docusate.[1] Ionic liquids with the vinylbenzene group were characterized through the C=C stretch (cs. 1630 cm^{-1}). New ionic liquid species were also characterized using ^{13}C NMR. NMR spectral assignments were made with the aid of COSY and HETCOR analysis.

2.1. Tributyl(vinylbenzyl)phosphonium Chloride ([P_{444VB}][Cl])

2.1.1. ^{1}H NMR (**Figure S1**)

^{1}H NMR (400 MHz, $CDCl_3$) δ 7.48 – 6.96 (m, 5H, H_{14}, H_{15}, H_{16}, H_{17}), 6.54 (dd, J = 17.6, 10.8 Hz, 1H, H_{18}), 5.64 (t, J = 17.5 Hz, 1H, H_{20}), 5.15 (dd, J = 11.0, 2.5 Hz, 1H, H_{19}), 2.77 – 1.83 (m, 10H, H_1, H_5, H_9, H_{13}), 1.33 (dq, J = 11.3, 6.5, 6.0 Hz, 13H, H_2, H_3, H_6, H_7, H_{10}, H_{11}), 1.03 – 0.54 (m, 9H, H_4, H_8, H_{12}).

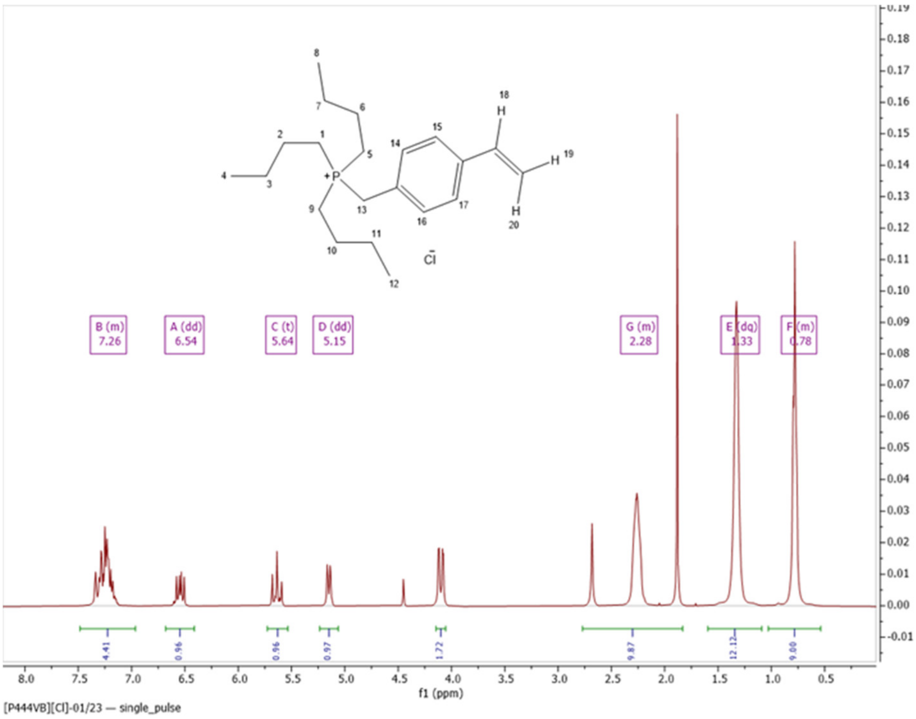

Figure S1: ^{1}H NMR Spectrum of [P_{444VB}][Cl]

2.1.2. MS (**Figure S2**)

MS (+ESI) (Acetonitrile): m/z 319.2550 [$C_{21}H_{36}P$] calc. 319.2555. (−ESI) (Acetonitrile): m/z Not Observed [Cl] calc. 34.9689.

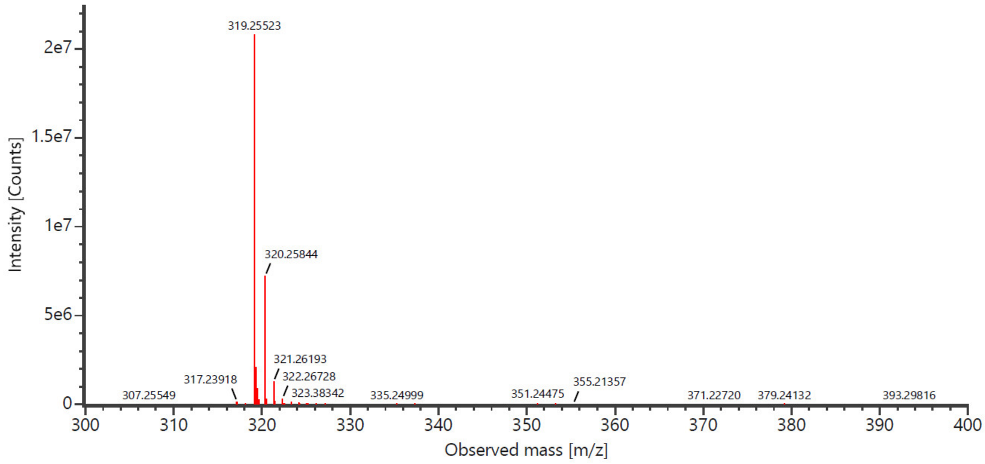

Figure S2: Mass Spectrum (+ESI) of [P_{444VB}] in [P_{444VB}][Cl]

2.2. Tributyl(octyl)phosphonium Bromide ([P_{4448}][Br])

2.2.1. ^{1}H NMR (**Figure S3**)

^{1}H NMR (400 MHz, $CDCl_3$) δ 2.42 – 2.31 (m, 3H, H_1, H_5, H_9), 2.35 – 2.27 (m, 1H, H_{13}), 1.93 (s, 1H, H_{14}), 1.47 (s, 2H, H_6, H_7), 1.53 – 1.44 (m, 2H, H_2, H_3), 1.44 (d, J = 7.6 Hz, 2H, H_{10}, H_{11}), 1.44 – 1.31 (m, 1H,), 1.29 – 1.13 (m, 2H, H_{15}, H_{16}), 1.17 (s, 3H, H_{17}, H_{18}, H_{19}), 0.92 – 0.81 (m, 5H, H_4,H_8), 0.85 – 0.74 (m, 2H, H_{12}, H_{20}).

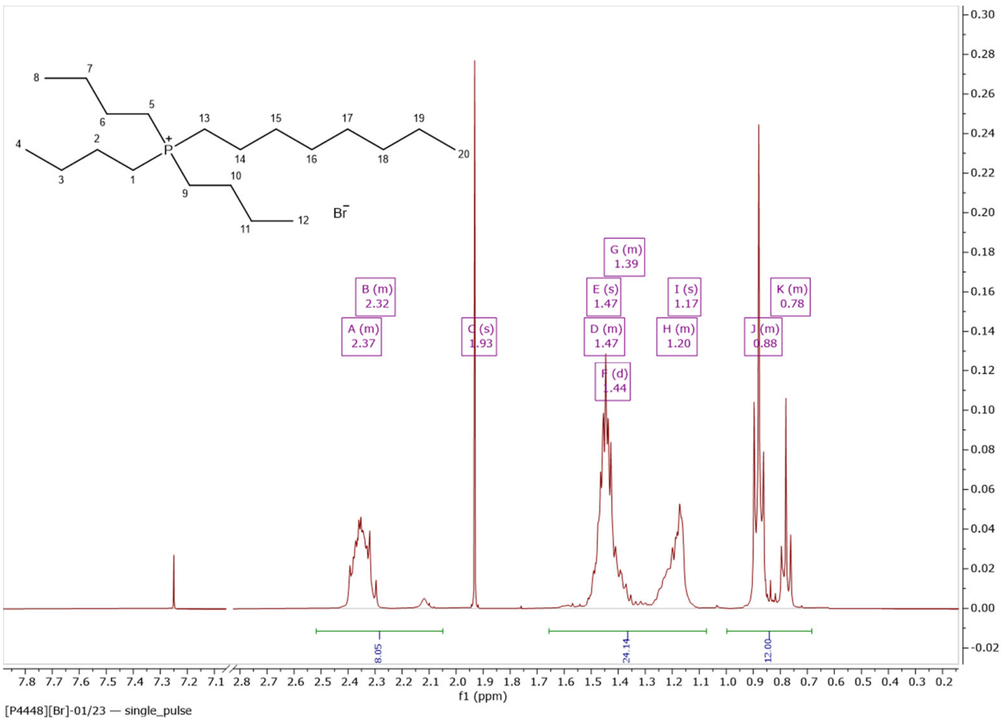

Figure S3: ^{1}H NMR Spectrum of [P_{4448}][Br]

2.2.2. MS (**Figure S4**)

MS (+ESI) (Acetonitrile): m/z 315.3177 [$C_{20}H_{44}P$] calc. 315.3181. (−ESI) (Acetonitrile): m/z Not Observed [Br] calc. 78.9183.

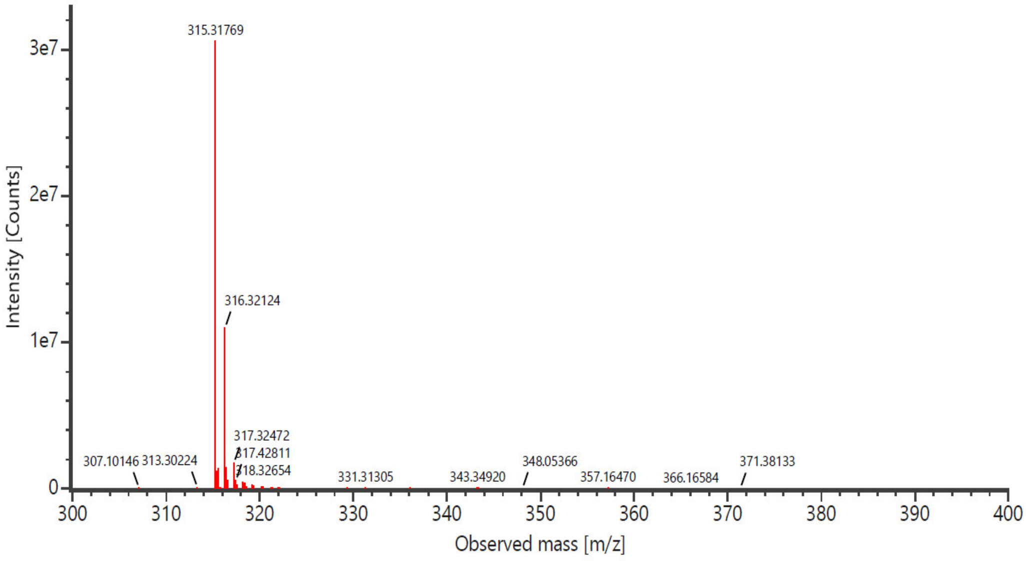

Figure S 4: Mass Spectrum (+ESI) of [P_{4448}] in [P_{4448}][Br]

2.3. *Tributyl(vinylbenzyl)phosphonium Docusate ([P_{444VB}] [AOT])*

2.3.1. ^{1}H NMR (**Figure S5**)

^{1}H NMR (400 MHz, $CDCl_3$) δ 7.43 – 7.25 (m, 4H, H_{14}, H_{15}, H_{16}, H_{17}), 6.67 (ddd, J = 17.6, 11.0, 2.1 Hz, 1H, H_{18}), 5.84 – 5.70 (m, 1H, H_{19}), 5.33 – 5.22 (m, 1H, H_{20}), 4.18 (dd, J = 11.4, 3.6 Hz, 1H), 4.08 – 3.84 (m, 5H, H_{21}, H_{22}, H_{31}), 3.27 (dd, J = 17.5, 11.4 Hz, 1H, H_{30}), 3.14 (dd, J = 17.5, 3.7 Hz, 1H, H_{30}), 2.29 – 2.13 (m, 4H, H_1, H_5), 1.99 (s, 1H, H_{23}), 1.60 (q, J = 5.7 Hz, 1H,

H_{32}), 1.56 – 1.19 (m, 18H, H_2, H_3, H_6, H_7, H_{10}, H_{11}, H_{26}, H_{27}, H_{28}, H_{24}, H_{35}, H_{36}, H_{37}), 1.25 (s, 7H, H_{24}, H_{35}, H_{36}, H_{37}), 0.96 – 0.76 (m, 17H, H_4, H_8, H_{12}, H_{25}, H_{29}, H_{34}, H_{38}).

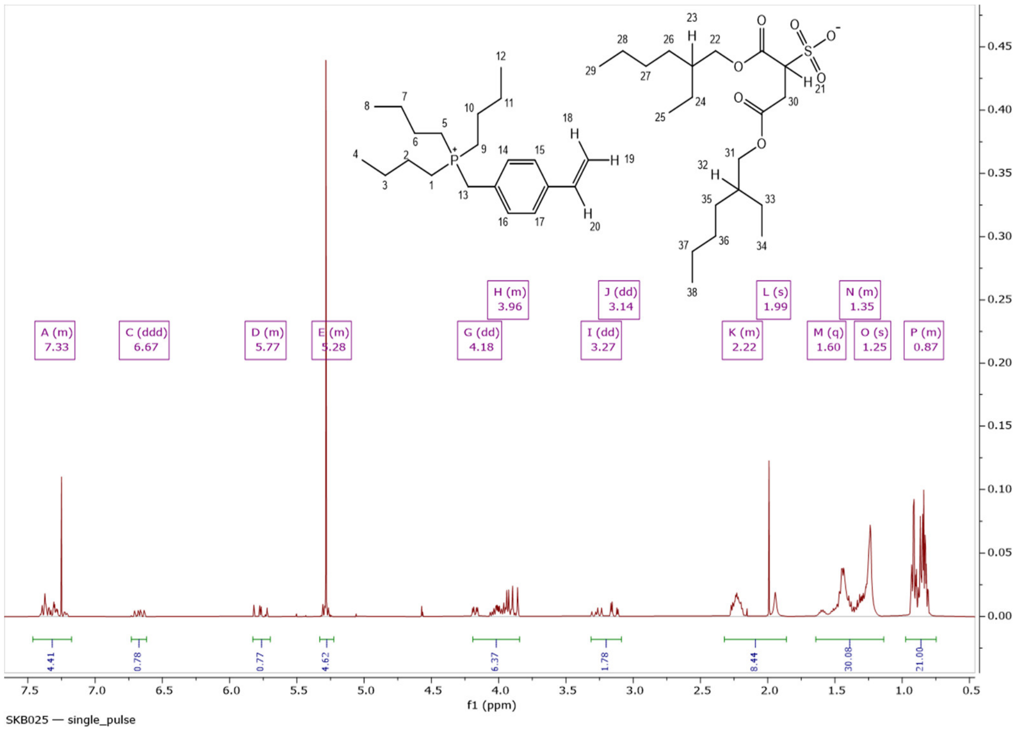

Figure S5: ^{1}H NMR Spectrum of [P_{444VB}][AOT]

2.3.2. FT-IR (**Figure S6**)

Table S3: FT-IR Identification of [P_{444VB}][AOT]

v/ cm^{-1}	Intensity	Appearance	Assignment	inference
2931	m	sh	C-H Stretch	Alkyl CH
1630	w	sh	C=C stretch	C=C conjugated with an aromatic ring, stronger than C=C
1512	w	sh	C=C stretch	Aromatic skeletal stretch
1731	s	sh	C=O	Aldehyde
1323	w	sh	S=O	Sulfonates
1158	s	sh	S=O	
853	s	sh	S-O	
990	s	sh	(RH)C=C(H_2)	Monosubstituted alkene
908	s	sh		

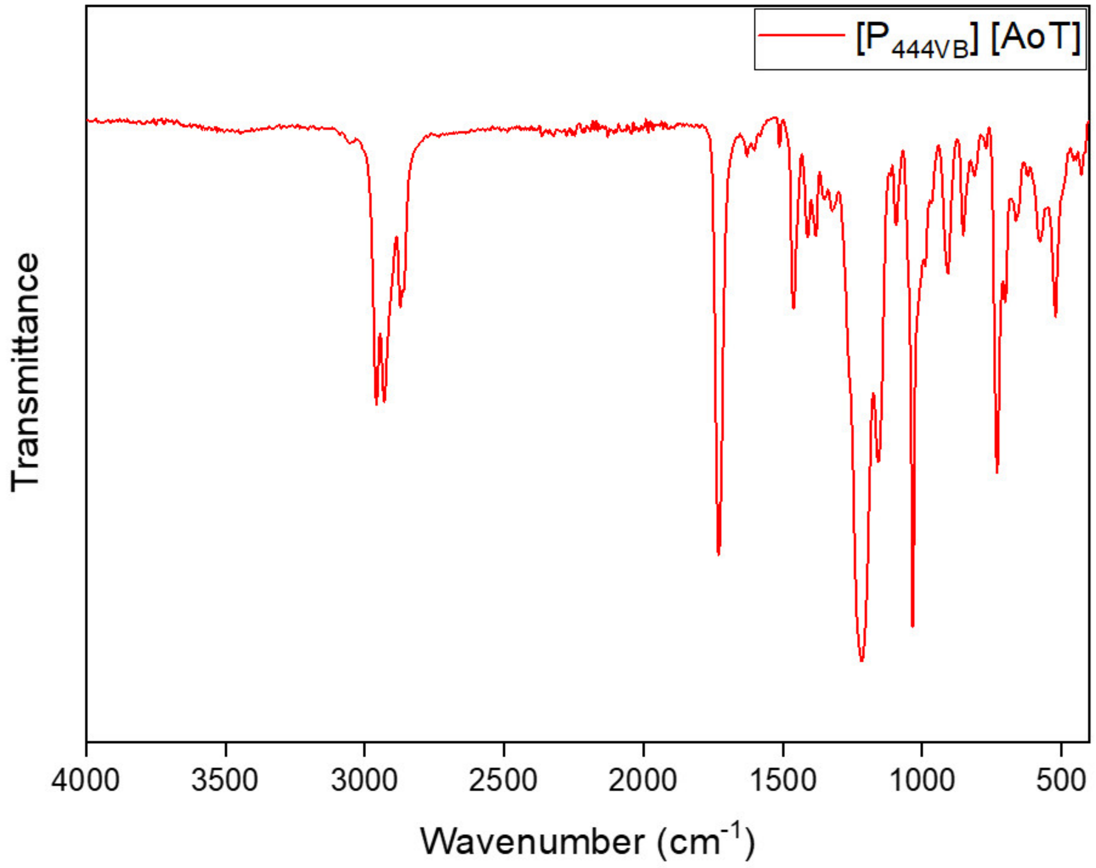

Figure S6: FT-IR Spectrum of [P_{444VB}][AOT]

2.3.3. MS (**Figure S7**, **Figure S8**)

MS (+ESI) (Acetonitrile): m/z 319.2548 [$C_{21}H_{36}P$] calc. 319.2555. (−ESI) (Acetonitrile): m/z 421.2267 [$C_{20}H_{37}O_7S$] calc. 421.2260.

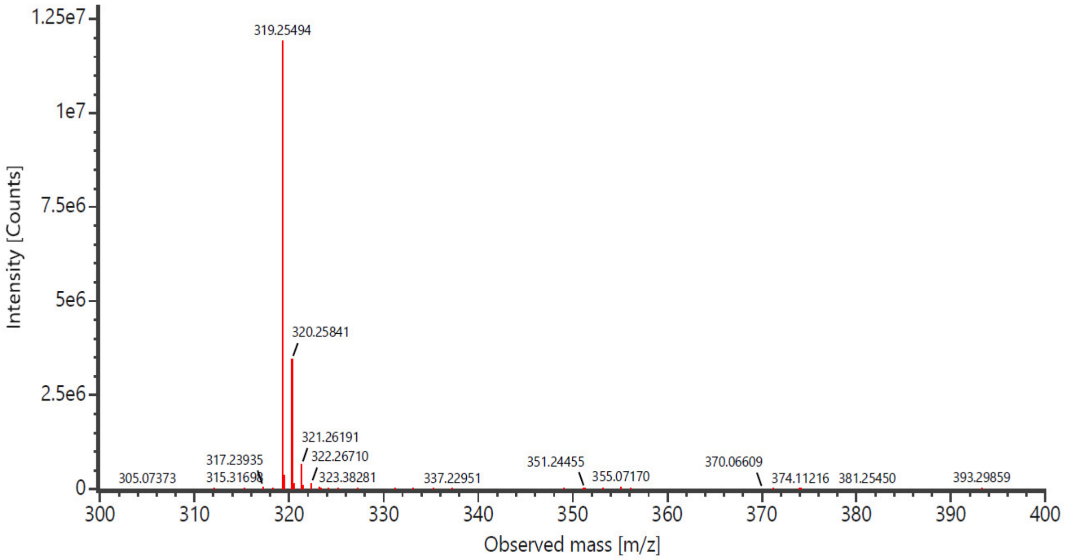

Figure S7: Mass Spectrum (+ESI) of [P_{444VB}] in [P_{444VB}][AOT]

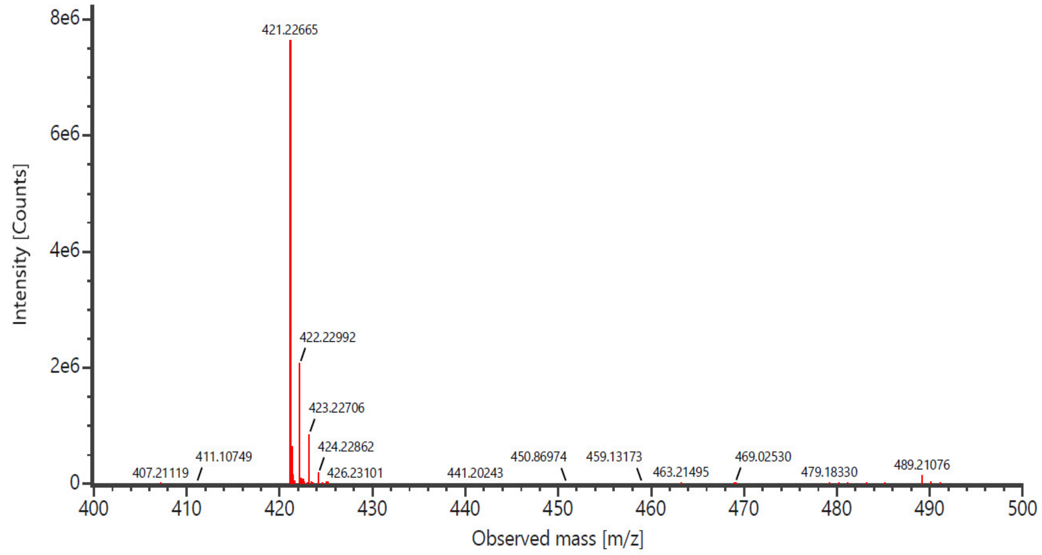

Figure S8: Mass Spectrum (−ESI) of [AOT] in [P_{444VB}][AOT]

2.4. Tributyl(octyl)phosphonium Docusate ([P_{4448}][AOT])

2.4.1. ^{1}H NMR (**Figure S9**)

^{1}H NMR (400 MHz, $CDCl_3$) δ 4.18 – 3.99 (m, 1H, H_{22}), 4.02 – 3.86 (m, 1H, H_{31}), 2.32 (s, 1H, H_{13}), 2.38 – 2.23 (m, 1H, H_1, H_5), 2.11 (s, 1H, H_9), 1.57 – 1.29 (m, 7H, H_{26}, H_{27}, H_{28}), 1.32 – 1.18 (m, 8H, H_{24}, H_{35}, H_{36}, H_{37}), 1.02 – 0.89 (m, 4H, H_{33}, H_2), 0.93 – 0.85 (m, 1H, H_{21}), 0.88 – 0.79 (m, 5H, H_4, H_8, H_{12}, H_{20}, H_{29}, H_{38}).

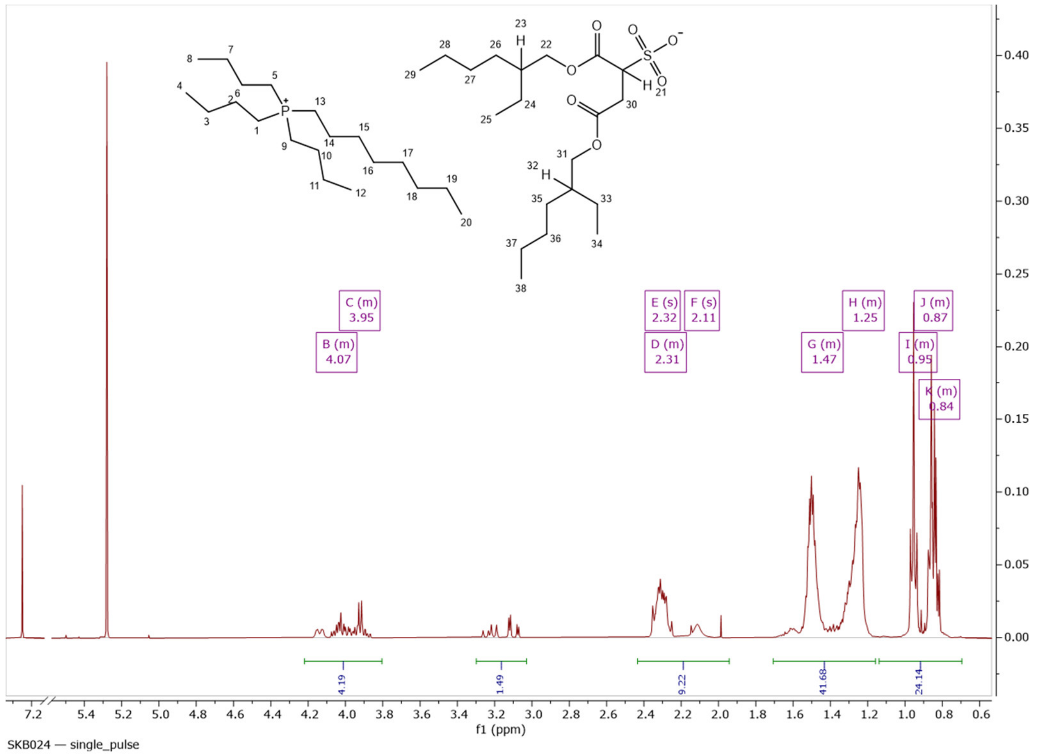

Figure S9: ^{1}H NMR Spectrum of [P_{4448}][AOT]

2.4.2. FT-IR (**Figure S10**)

Table S4: FT-IR Identification of [P_{4448}][AOT]

v/ cm $^{-1}$	Intensity	Appearance	Assignment	inference
2926	s	sh	C-H Stretch	Alkyl CH
1680	s	sh	C=O	Aldehyde
1313	w	sh	S=O	Sulfonates
1160	w	sh	S=O	
810	s	sh	S-O	

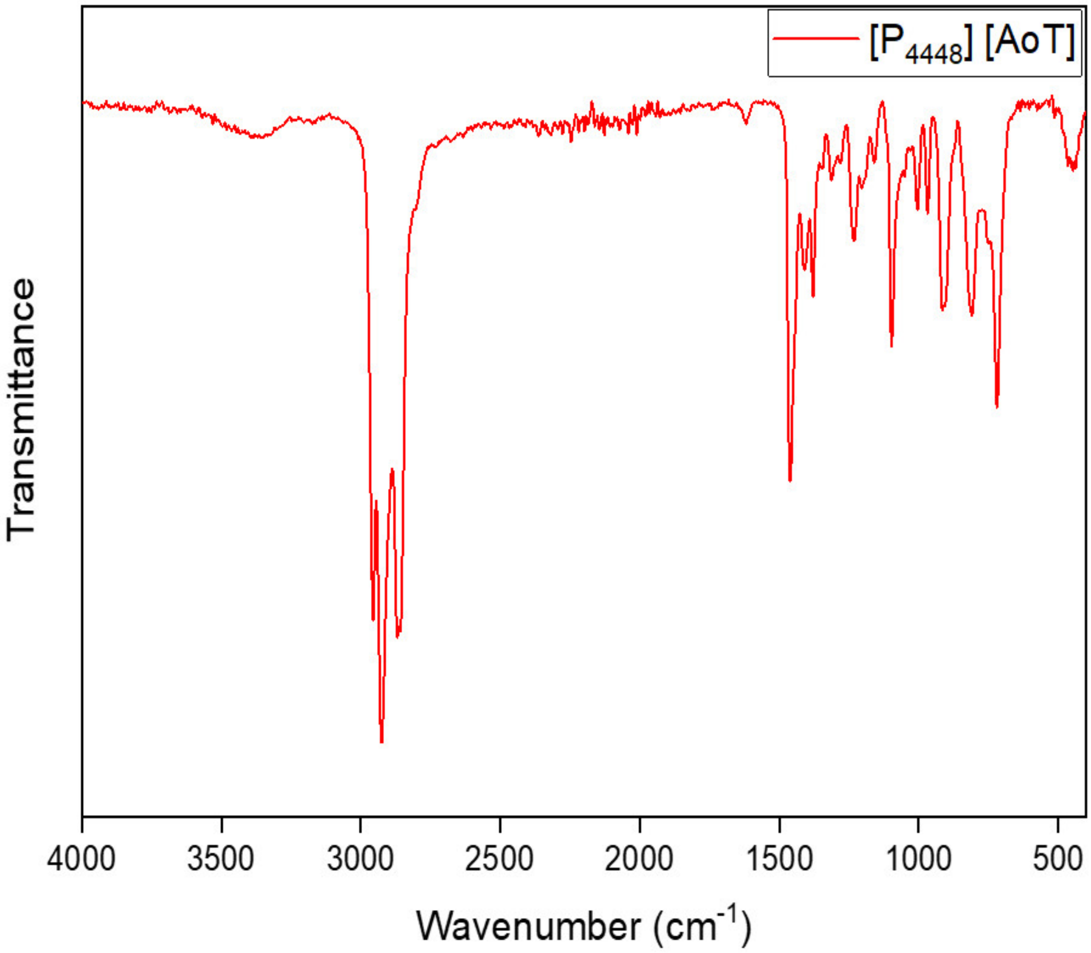

Figure S10: FT-IR Spectrum of [P_{4448}][AOT]

2.4.3. MS (**Figure S11**, **Figure S12**)

MS (+ESI) (Acetonitrile): m/z 315.3175 [$C_{20}H_{44}P$] calc. 315.3181. (−ESI) (Acetonitrile): m/z 421.2268 [$C_{20}H_{37}O_7S$] calc. 421.2260.

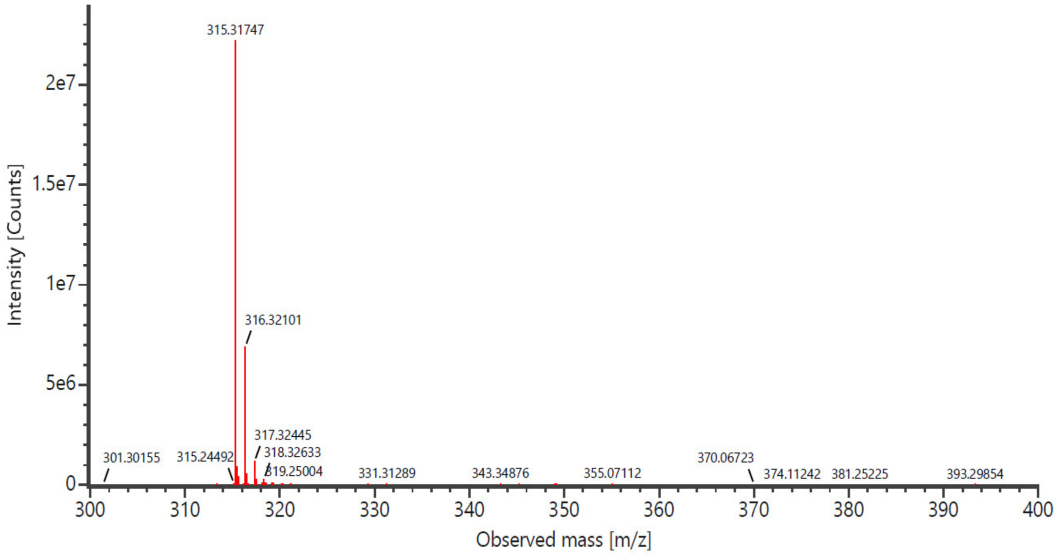

Figure S11: Mass Spectrum (+ESI) of [P_{4448}] in [[P_{4448}][AOT]

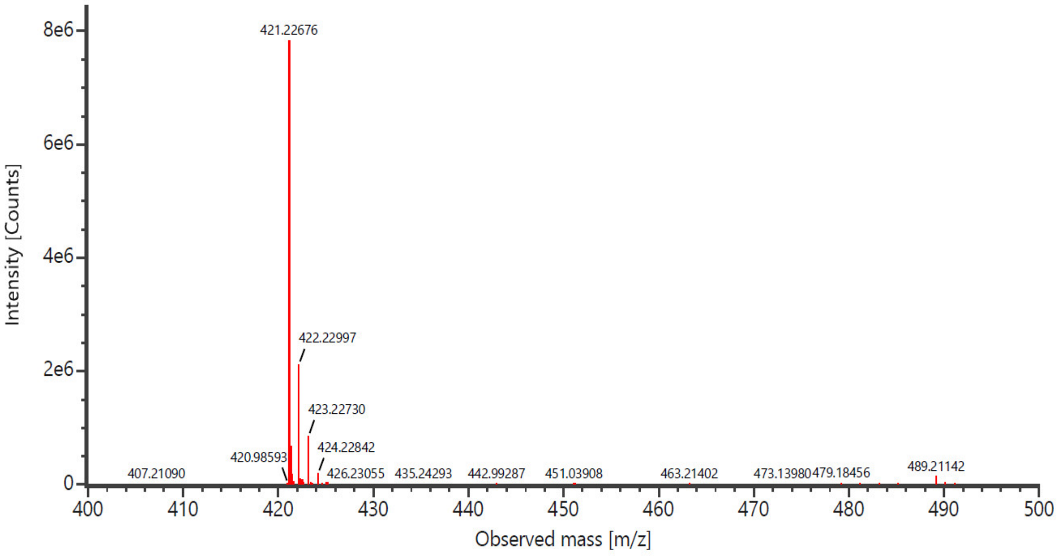

Figure S12: Mass Spectrum (−ESI) of [AOT] in [[P_{4448}][AOT]

2.5. Trioctyl(vinylbenzyl)phosphonium Chloride ([P_{888VB}][Cl])

2.5.1. ^{1}H NMR (**Figure S13**)

^{1}H NMR (62 MHz, CDCl3) δ 7.23 (q, J = 3.4, 2.4 Hz, 1H, H_{26}, H_{27}, H_{28}, H_{29}), 2.21 (dd, J = 15.0, 7.3 Hz, 1H, H_1, H_9, H_{18}, H_{25}), 1.23 (d, J = 5.2 Hz, 2H, H_2, H_3, H_4, H_5, H_6, H_7, H_{10}, H_{11}, H_{12}, H_{13}, H_{14}, H_{15}, H_{18}, H_{19}, H_{20}, H_{21}, H_{22}, H_{23}), 0.90 (d, J = 22.4 Hz, 5H, H_8, H_{16}, H_{24}).

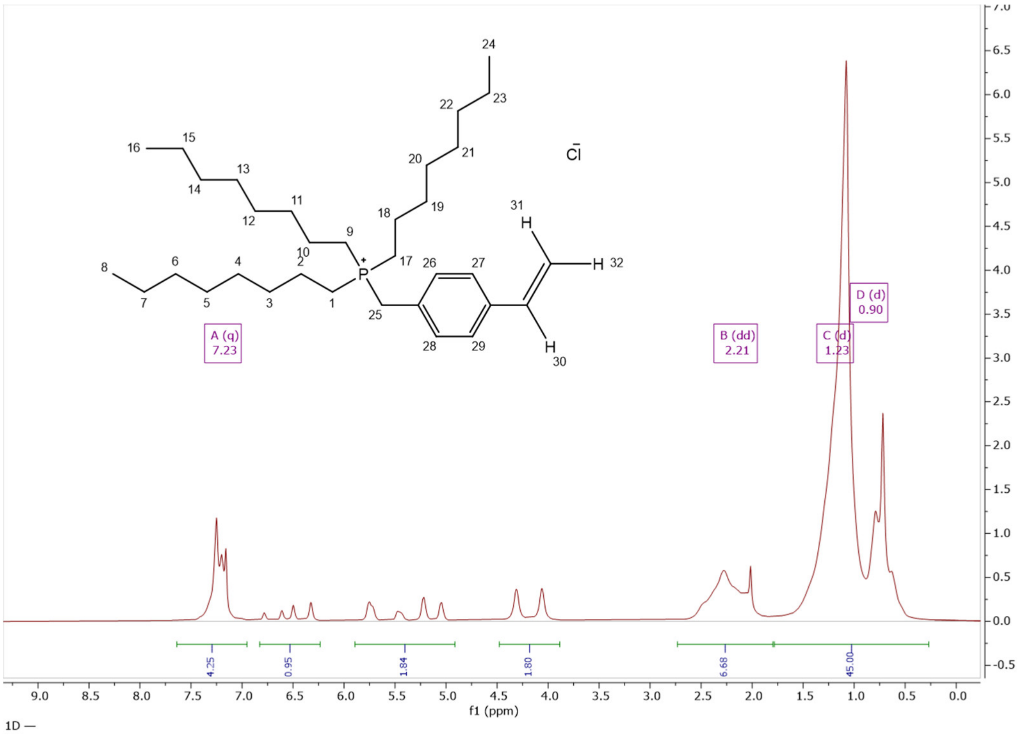

Figure S13: ^{1}H NMR Spectrum of [P_{888VB}][Cl]

2.5.2. FT-IR (**Figure S14**)

Table S5: FT-IR Classification of [P_{888VB}][Cl]

v/ cm $^{-1}$	Intensity	Appearance	Assignment	inference
2923	s	sh	C-H Stretch	Alkyl CH
1629	w	sh	C=C stretch	C=C conjugated with an aromatic ring, stronger than C=C
1511	w	sh	C=C stretch	Aromatic skeletal stretch
989	s	sh	(RH)C=C(H_2)	Monosubstituted alkene
904	s	sh		

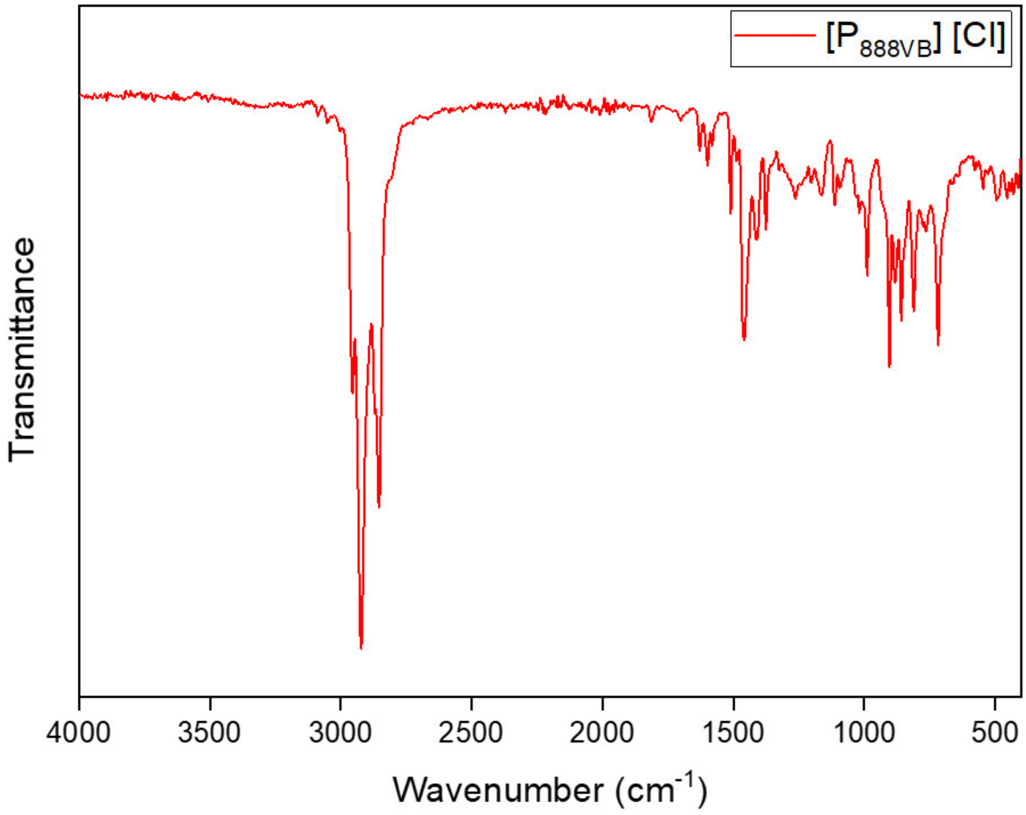

Figure S14: FT-IR Spectrum of [P_{888VB}][Cl]

2.5.3. MS (**Figure S15**)

MS (+ESI) (Acetonitrile): m/z 487.4424 [$C_{33}H_{60}P$] calc. 487.4433. (−ESI) (Acetonitrile): m/z Not Observed [Cl] calc. 34.9689.

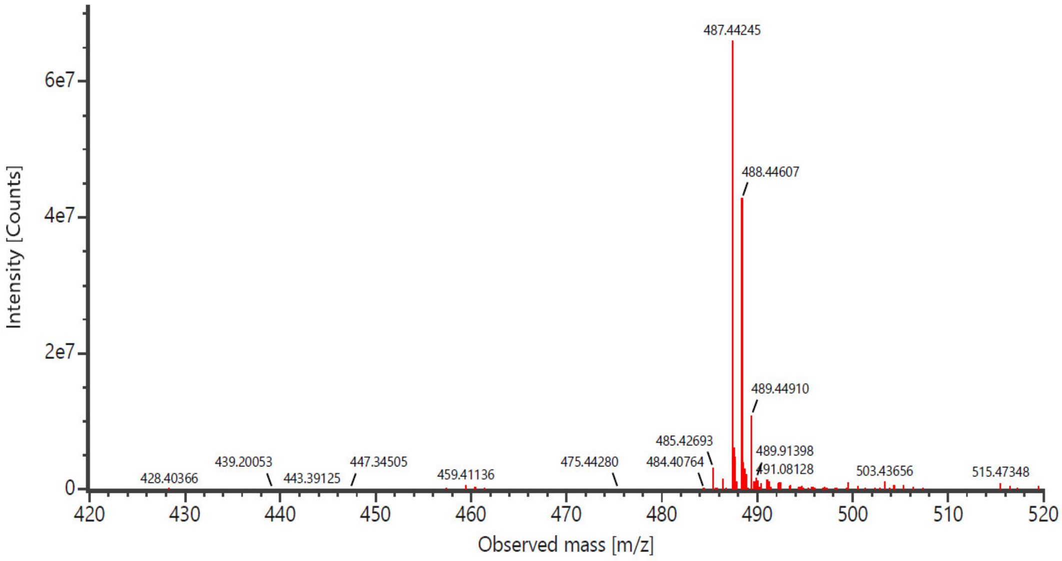

Figure S15: Mass Spectrum (+ESI) of [P_{888VB}][Cl]

2.6. Trioctyl(tetradecyl)phosphonium Bromide ([P_{88814}][Br])

2.6.1. ^{1}H NMR (**Figure S16**)

^{1}H NMR (62 MHz, CDCl3) δ 2.78 – 1.94 (m, 0H, H_1, H_9, H_{17}, H_{25}), 1.56 – 0.51 (m, 4H, H_2, H_3, H_4, H_5, H_6, H_7, H_8, H_{10}, H_{11}, H_{12}, H_{13}, H_{14}, H_{15}, H_{16}, H_{18}, H_{19}, H_{20}, H_{21}, H_{22}, H_{23}, H_{24}, H_{26}, H_{27}, H_{28}, H_{29}, H_{30}, H_{31}, H_{32}, H_{33}, H_{34}, H_{35}, H_{36}, H_{37}).

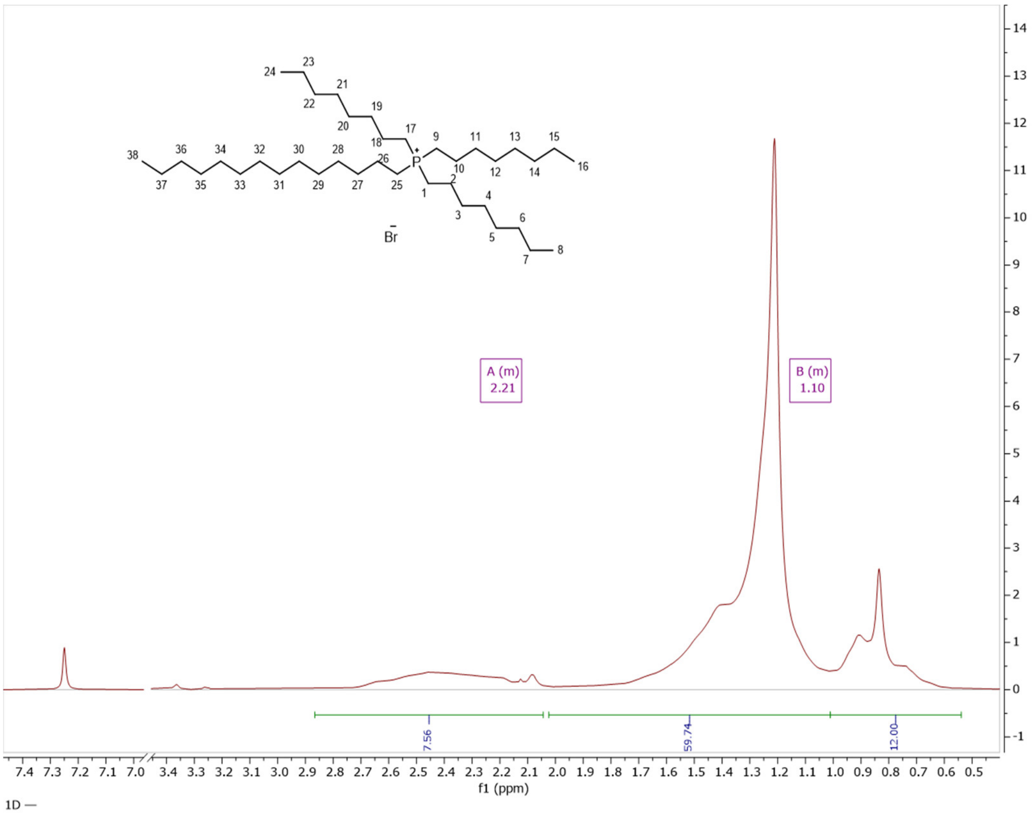

Figure S16: ^{1}H NMR Spectrum of [P_{88814}][Br]

2.6.2. FT-IR (**Figure S17**)

Table S6: FT-IR Classification of [P_{88814}][Br]

v/ cm $^{-1}$	Intensity	Appearance	Assignment	inference
2921	s	sh	C-H Stretch	Alkyl CH

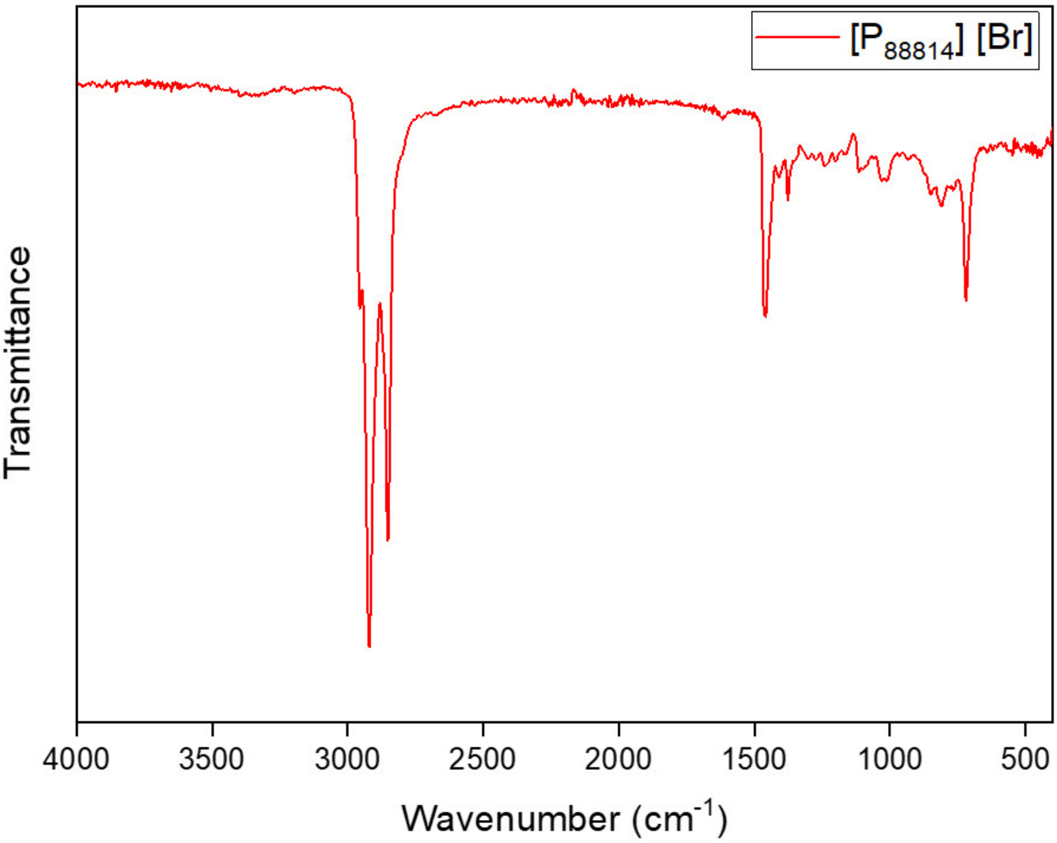

Figure S17: FT-IR Spectrum of [P_{88814}][Br]

2.6.3. MS (**Figure S18**)

MS (+ESI) (Acetonitrile): m/z 567.5990 [$C_{38}H_{80}P$] calc. 567.5998. (−ESI) (Acetonitrile): m/z Not Observed [Br] calc. 78.9183.

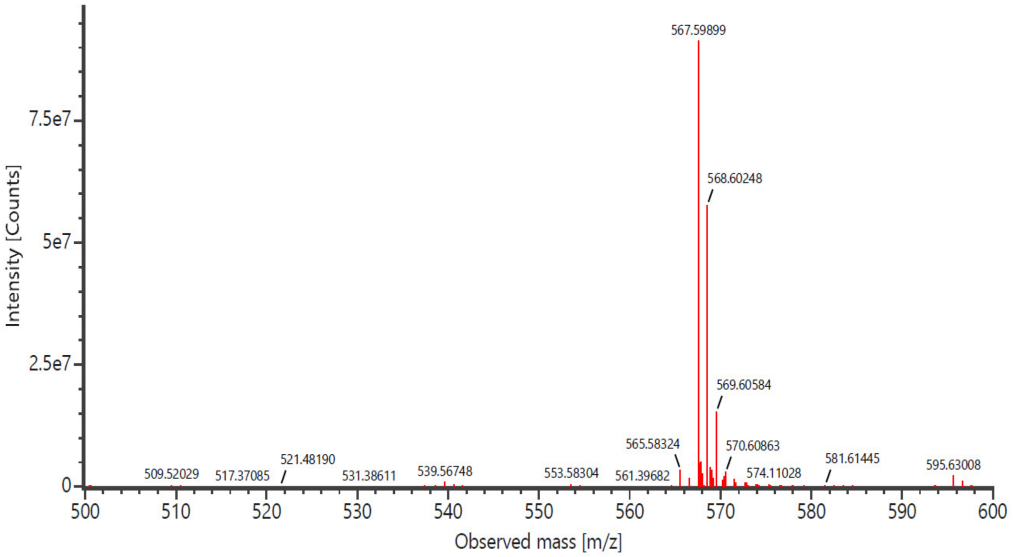

Figure S18: Mass Spectrum (+ESI) of [P_{88814}] in [P_{88814}][Br]

2.7. Trioctyl(vinylbenzyl)phosphonium Docusate ([P_{888VB}] [AOT])

2.7.1. ^{1}H NMR (**Figure S19**)

^{1}H NMR (400 MHz, $CDCl_3$) δ 7.43 – 7.25 (m, 4H, H_{14}, H_{15}, H_{16}, H_{17}), 6.67 (ddd, J = 17.6, 11.0, 2.1 Hz, 1H, H_{18}), 5.84 – 5.70 (m, 1H, H_{19}), 5.33 – 5.22 (m, 1H, H_{20}), 4.18 (dd, J = 11.4, 3.6 Hz, 1H), 4.08 – 3.84 (m, 5H, H_{21}, H_{22}, H_{31}), 3.27 (dd, J = 17.5, 11.4 Hz, 1H, H_{30}), 3.14 (dd, J = 17.5, 3.7 Hz, 1H, H_{30}), 2.29 – 2.13 (m, 4H, H_{1}, H_{5}), 1.99 (s, 1H, H_{23}), 1.60 (q, J = 5.7 Hz, 1H, H_{32}), 1.56 – 1.19 (m, 18H, H_{2}, H_{3}, H_{6}, H_{7}, H_{10}, H_{11}, H_{26}, H_{27}, H_{28}, H_{24}, H_{35}, H_{36}, H_{37}), 1.25 (s, 7H, H_{24}, H_{35}, H_{36}, H_{37}), 0.96 – 0.76 (m, 17H, H_{4}, H_{8}, H_{12}, H_{25}, H_{29}, H_{34}, H_{38}).

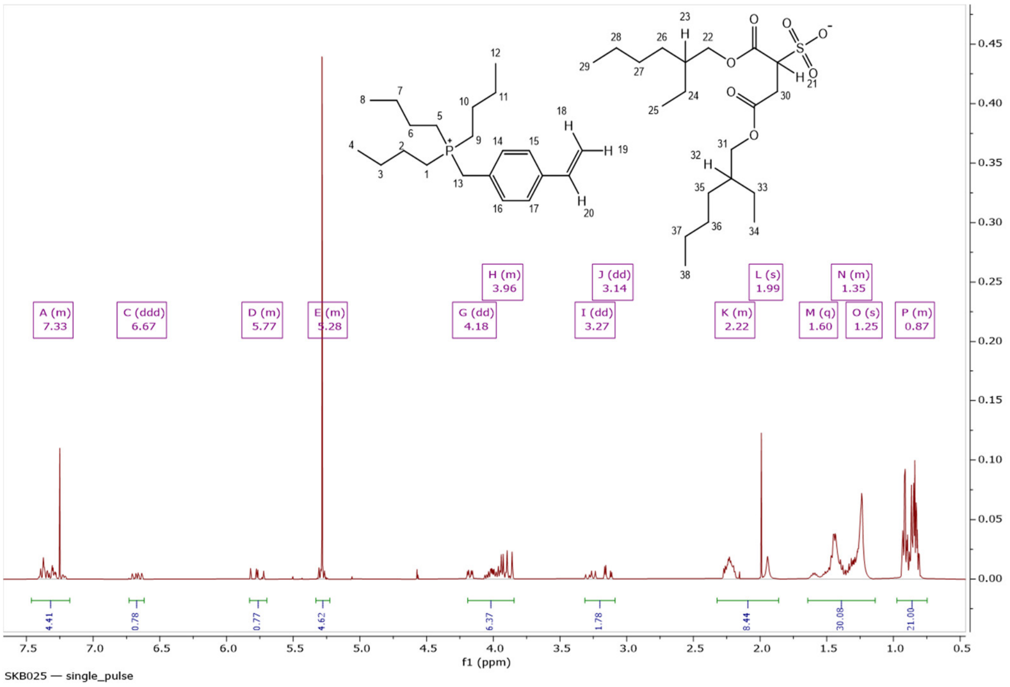

Figure S19: ^{1}H NMR Spectrum of in [P_{888VB}][AOT]

2.7.2. ^{13}C NMR (**Figure S20**)

^{13}C NMR (101 MHz, $CDCl_3$*D*) δ 171.76, C_{12} 169.16, C_{8} 135.80 C_{61}, C_{64}, 130.36, 130.30, 129.63, 129.34, 129.29, 127.83, 127.17, 127.13, 126.09, 115.36, 114.88 C_{65}, 67.57, 67.49, 66.89, 61.97, 53.40, 38.66, 38.64, 38.52, 38.46, 34.36, 31.66, 30.84, 30.70, 30.29, 30.25, 30.07, 30.01, 28.91, 28.87, 28.81, 27.01, 26.85, 26.56, 23.62, 23.38, 22.96, 22.93, 22.55, 21.80, 21.75, 18.85, 18.39, 14.05, 14.01, 10.92, 10.87, 10.76, 10.73.

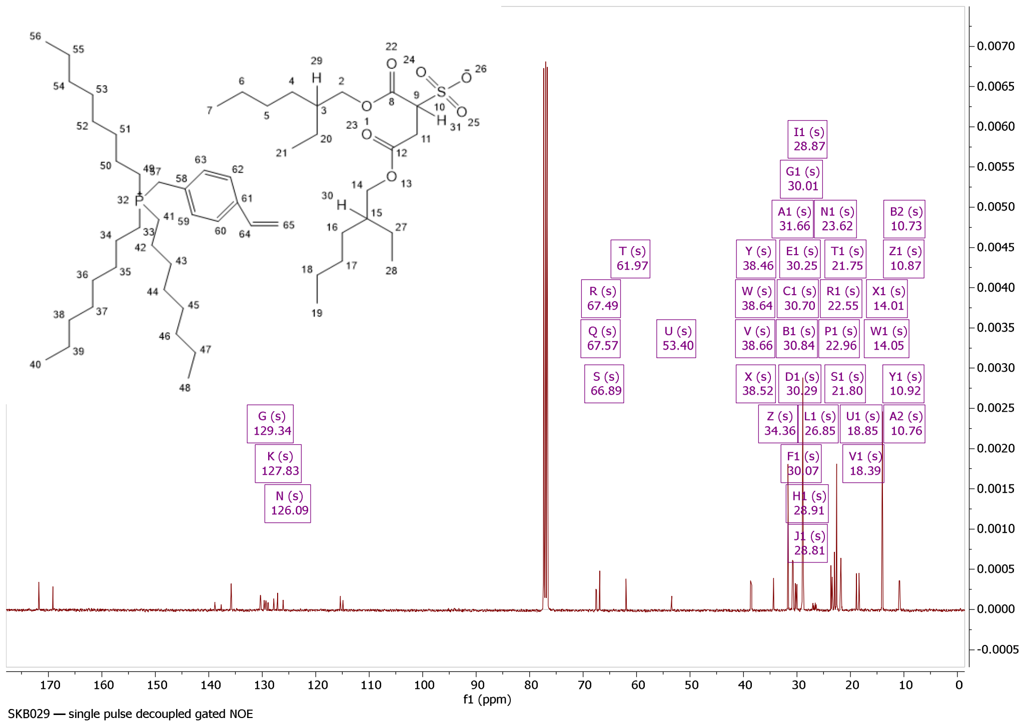

Figure S20: ^{13}C NMR Spectrum of in $[P_{888VB}][AOT]$

2.7.3. COSY (**Figure S21**)

1H NMR (400 MHz, $CDCl_3$) δ 5.66, 5.18, 6.61, 6.56, 3.29, 3.12, 1.48, 3.08, 4.12, 3.25, 4.12, 1.38, 3.94, 2.12, 1.44, 0.78, 1.24

1H NMR (400 MHz, $CDCl_3$) δ 6.61, 6.60, 5.72, 5.23, 4.11, 4.10, 3.89, 3.25, 3.24, 3.08, 3.08, 2.14, 1.52, 1.35, 1.28, 1.27, 0.79.

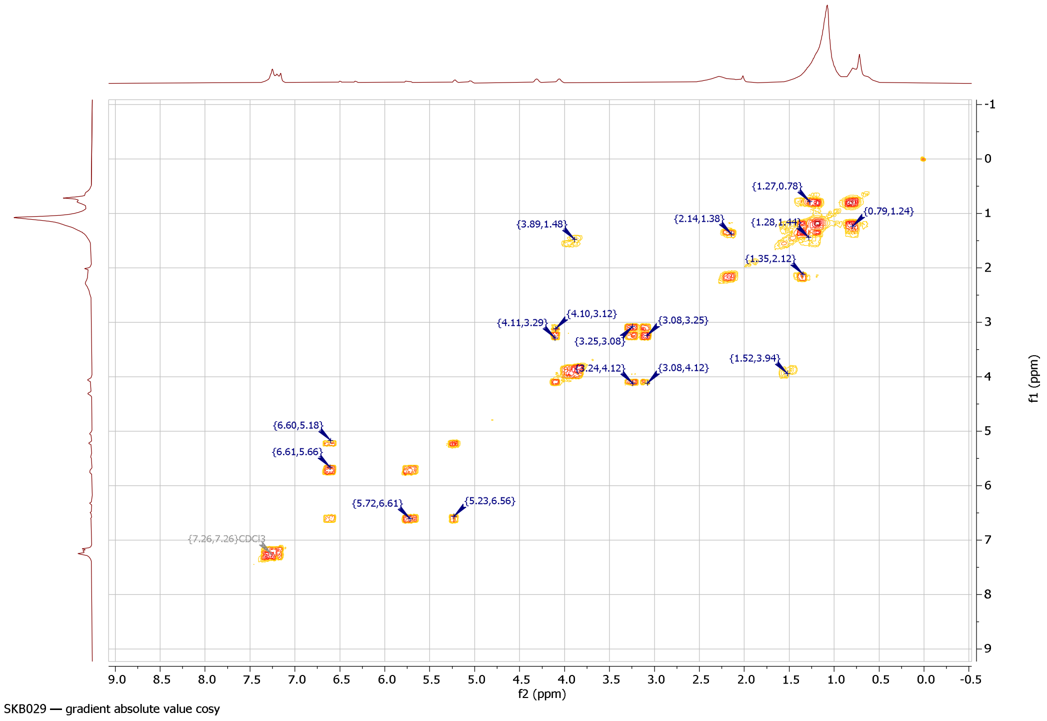

Figure S21: COSY Spectrum of in $[P_{888VB}][AOT]$

2.7.4. FT-IR (**Figure S22**)

Table S7: FT-IR Classification of [P_{888VB}][AOT]

v/ cm $^{-1}$	Intensity	Appearance	Assignment	inference
2926	s	sh	C-H Stretch	Alkyl CH
1630	w	sh	C=C stretch	C=C conjugated with an aromatic ring, stronger than C=C
1512	w	sh	C=C stretch	Aromatic skeletal stretch
1732	s	sh	C=O	Aldehyde
1325	w	sh	S=O	Sulfonates
1155	s	sh	S=O	
856	s	sh	S-O	
989	s	sh	(RH)C=C(H_2)	Monosubstituted alkene
906	s	br		

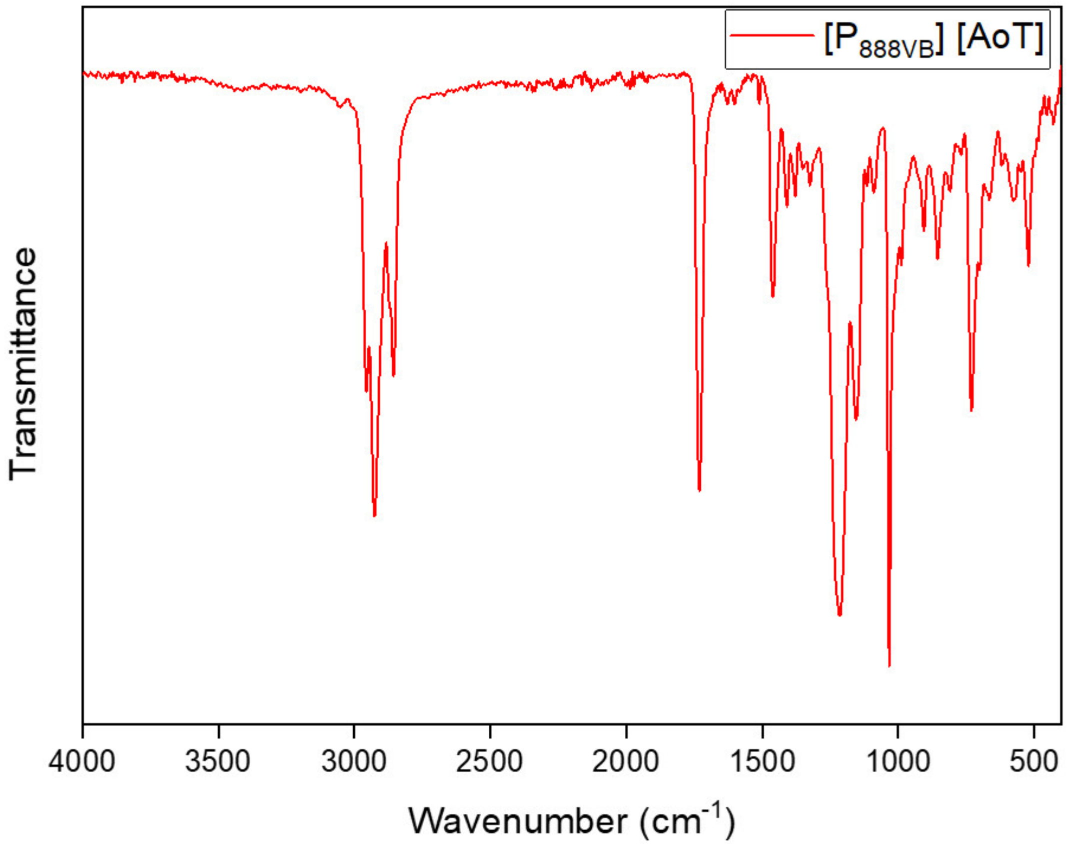

Figure S22: FT-IR Spectrum of [P_{888v}B][AOT]

2.7.5. MS (**Figure S23**, **Figure S24**)

MS (+ESI) (Acetonitrile): m/z 487.4426 [$C_{33}H_{60}P$] calc. 487.4433. (−ESI) (Acetonitrile): m/z 421.2263 [$C_{20}H_{37}O_7S$] calc. 421.2260

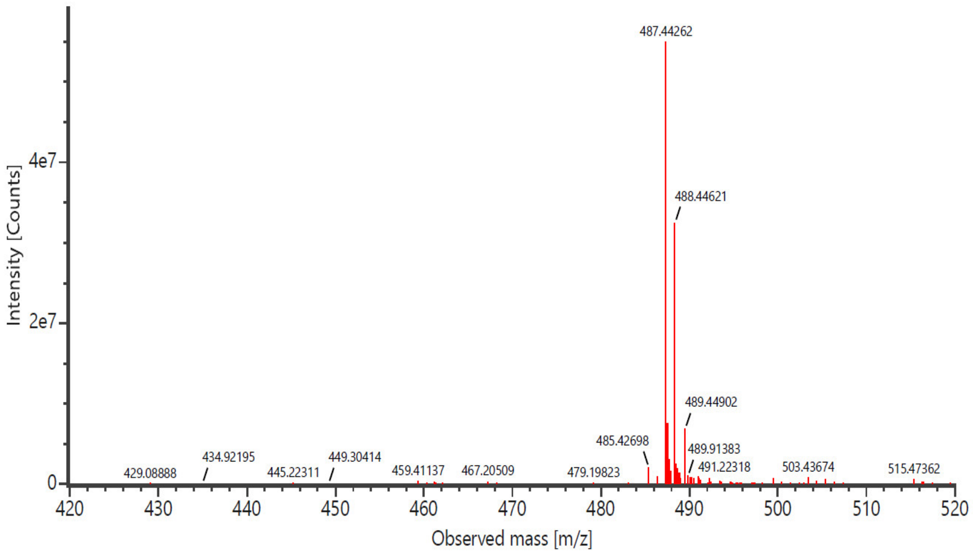

Figure S23: Mass Spectrum (+ESI) of [P_{888VB}] in [P_{888VB}][AOT]

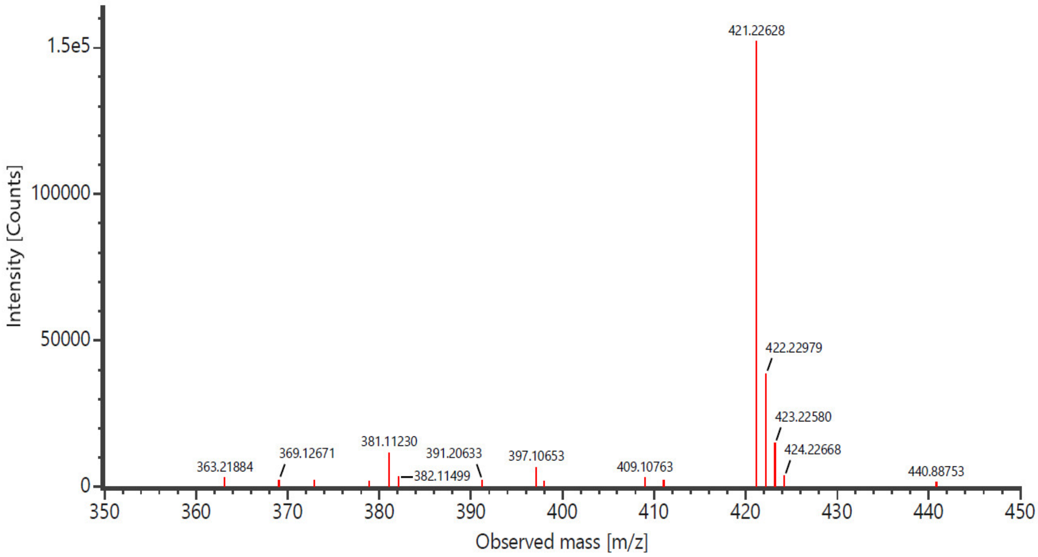

Figure S24: Mass Spectrum (−ESI) of [AoT] in [P_{888VB}][AOT]

2.8. Trioctyl(tetradecyl)phosphonium Docusate ([P_{88814}] [AOT])

2.8.1. ^{1}H NMR (**Figure S25**)

^{1}H NMR (400 MHz, $CDCl_3$) δ 4.20 – 3.85 (m, 4H, H_{40}, H_{49}), 3.29 – 3.06 (m, 1H, H_{39}), 2.36 – 2.22 (m, 7H, H_1, H_9, H_{17}, H_{25}), 2.02 (d, J = 27.5 Hz, 3H, H_{41}, H_{50}), 1.68 – 1.15 (m, 79H, H_2, H_3, H_4 ,H_5, H_6, H_7, H_{10}, H_{11}, H_{12}, H_{13}, H_{14}, H_{15}, H_{18}, H_{19}, H_{20}, H_{21}, H_{22}, H_{23}, H_{26}, H_{27}, H_{28}, H_{29}, H_{30}, H_{31}, H_{32}, H_{33}, H_{34}, H_{35}, H_{36}, H_{37}, $H_{42,}$ H_{44}, H_{45}, H_{46}, $H_{51,}$ H_{53}, H_{54}, H_{55}), 0.93 – 0.79 (m, 24H, H_8, H_{16}, H_{24}, H_{38}, H_{43}, H_{47}, H_{52}, H_{56}).

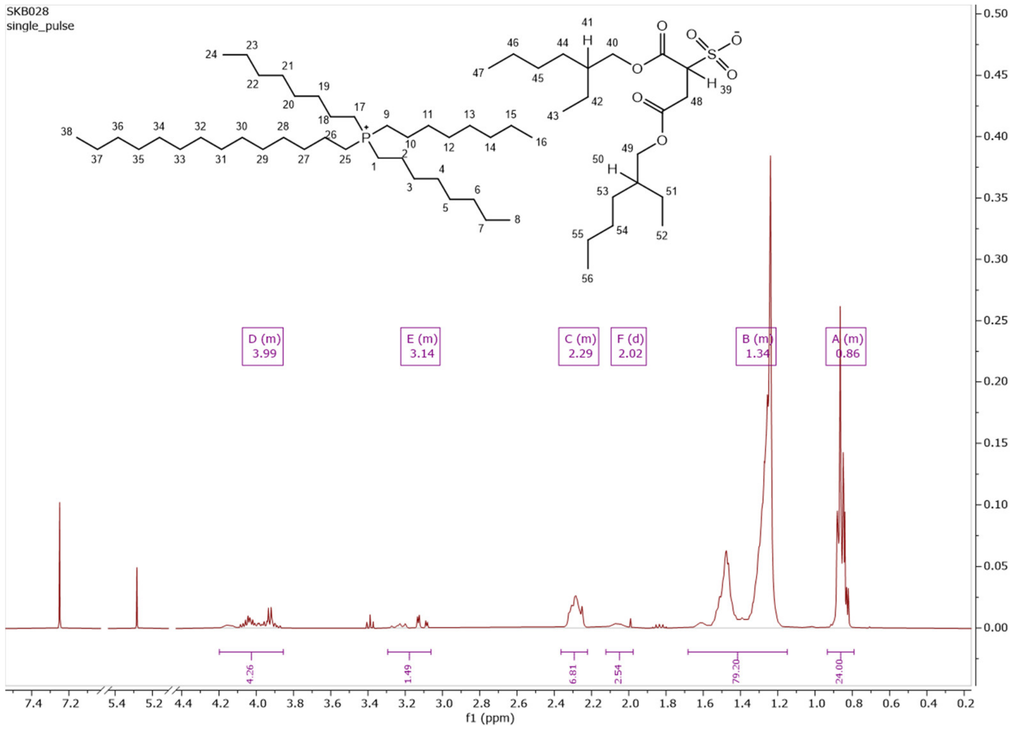

Figure S25: ^{1}H NMR Spectrum of [P_{88814}][AOT]

2.8.2. ^{13}C NMR (**Figure S26**)

^{13}C NMR (101 MHz, $CDCl_3$) δ 171.72, C_{51}, 169.27, C_{47}, 67.02, C_{41}, C_{53}, 61.73, C_{48}, 38.64, 38.52, 38.46, 34.05, 31.90, 31.69, 30.85, 30.70, 30.28, 30.25, 30.09, 30.04, 29.67, 29.63, 29.53, 29.34, 29.30, 29.02, 28.95, 28.87, 23.61, 23.40, 22.98, 22.95, 22.67, 22.58, 21.94, 21.89, 19.29, 18.83, 14.09, 14.07, 14.03, 10.91, 10.87, 10.82, 10.77.

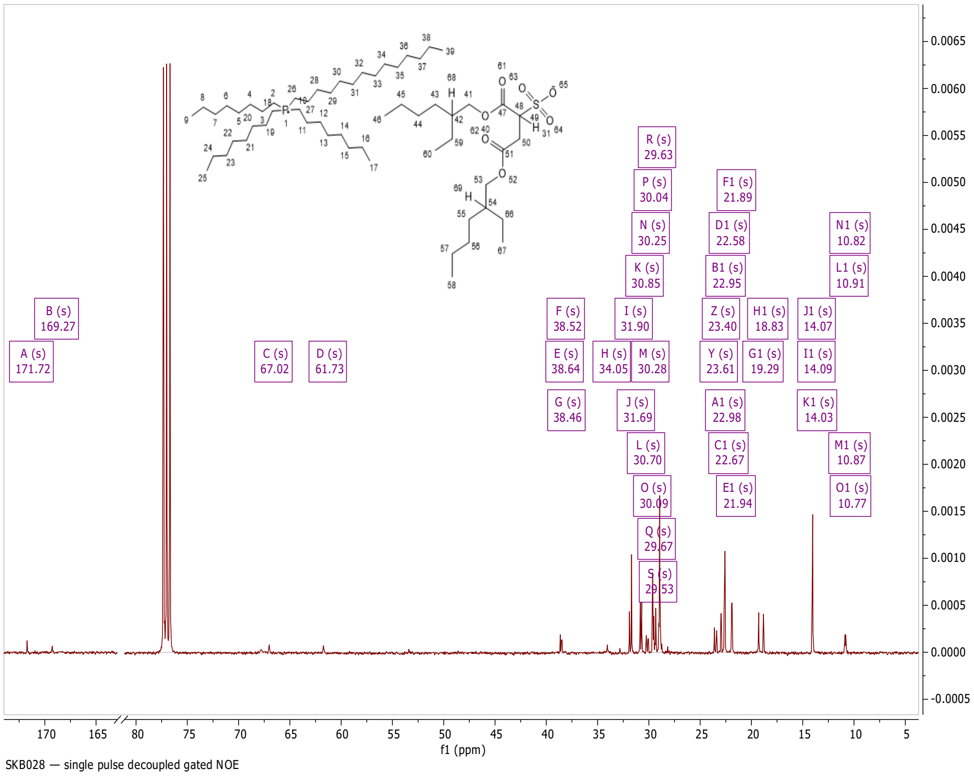

Figure S26: ^{13}C NMR Spectrum of [P_{88814}][AOT]

2.8.3. COSY (**Figure S27**)

^{1}H NMR (400 MHz, $CDCl_3$) δ 3.17, 1.62, 1.53, 1.85, 3.12, 4.15, 3.23, 1.49, 1.40, 3.41, 4.07, 2.29, 3.94, 1.27, 0.89, 1.85, 1.48, 0.88, 1.29, 1.27

^{1}H NMR (400 MHz, $CDCl_3$) δ 4.17, 4.07, 3.92, 3.38, 3.29, 3.21, 3.15, 2.28, 1.87, 1.84, 1.64, 1.55, 1.52, 1.49, 1.42, 1.40, 1.30, 1.29, 0.87, 0.74.

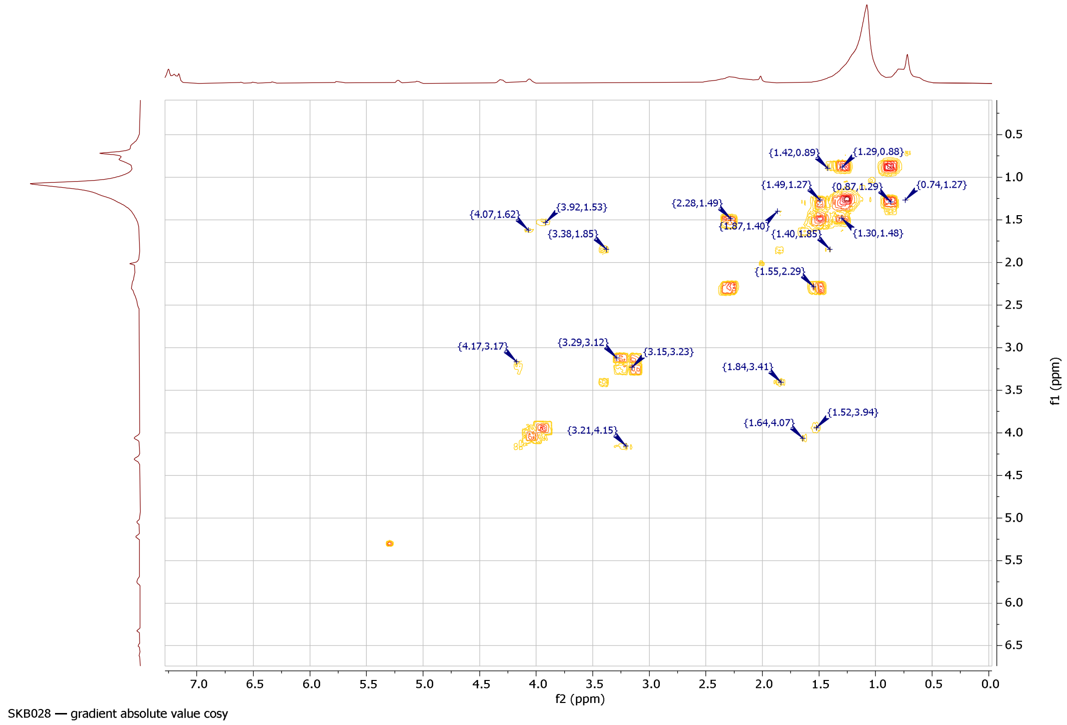

Figure S27: COSY Spectrum of [P_{88814}][AOT]

2.8.4. FT-IR (**Figure S28**)

Table S8: FT-IR Classification of [P_{88814}][AOT]

v/ cm -1	Intensity	Appearance	Assignment	inference
2923	s	sh	C-H Stretch	Alkyl CH
1733	s	sh	C=O	Aldehyde
1352	w	sh	S=O	Sulfonates
1157	s	sh	S=O	
771	s	sh	S-O	

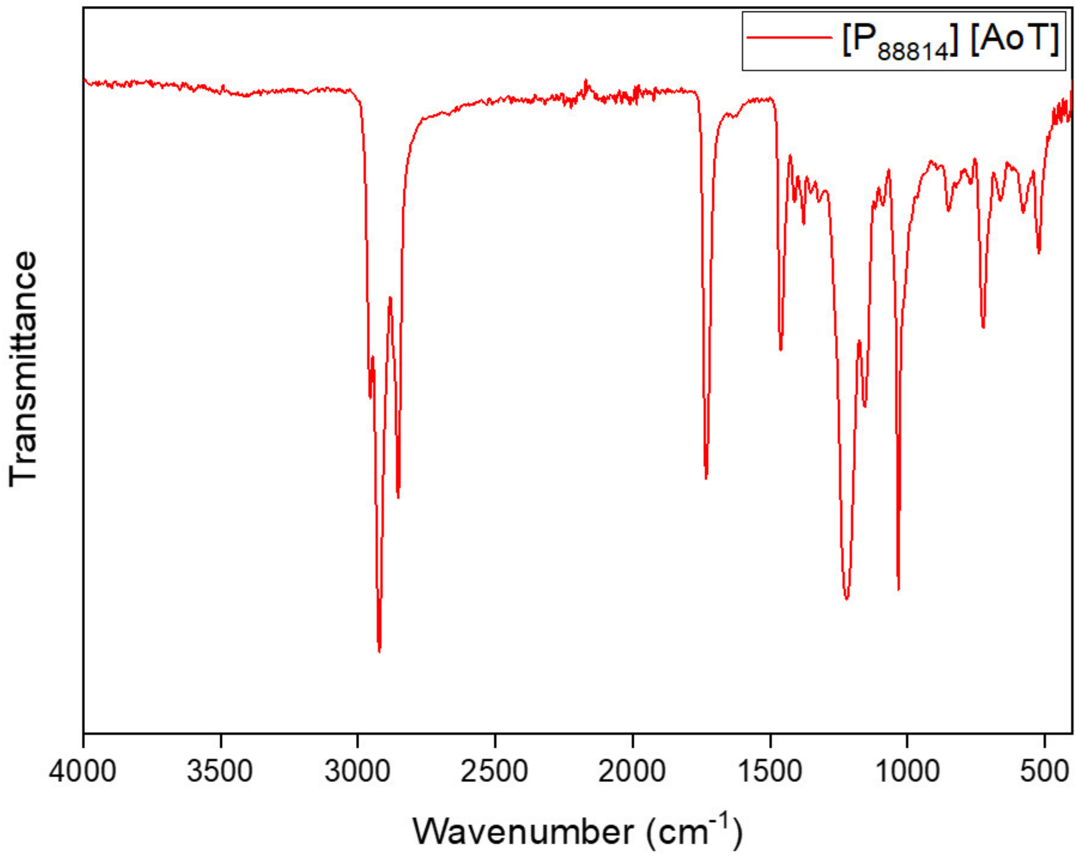

Figure S28: FT-IR Spectrum of [P_{88814}][AOT]

2.8.5. MS (**Figure S29**, **Figure S30**)

MS (+ESI) (Acetonitrile): m/z 567.5989 [$C_{38}H_{80}P$] calc. 567.5998. (−ESI) (Acetonitrile): m/z 421.2265 [$C_{20}H_{37}O_7S$] calc. 421.2260.

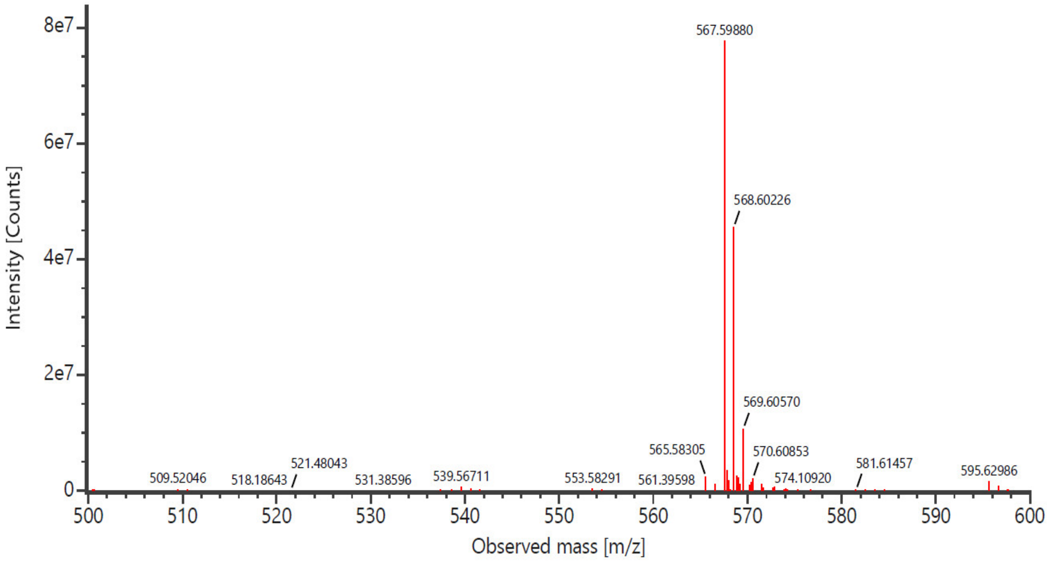

Figure S29: Mass Spectrum (+ESI) of [P_{88814}] in [P_{88814}] [AOT]

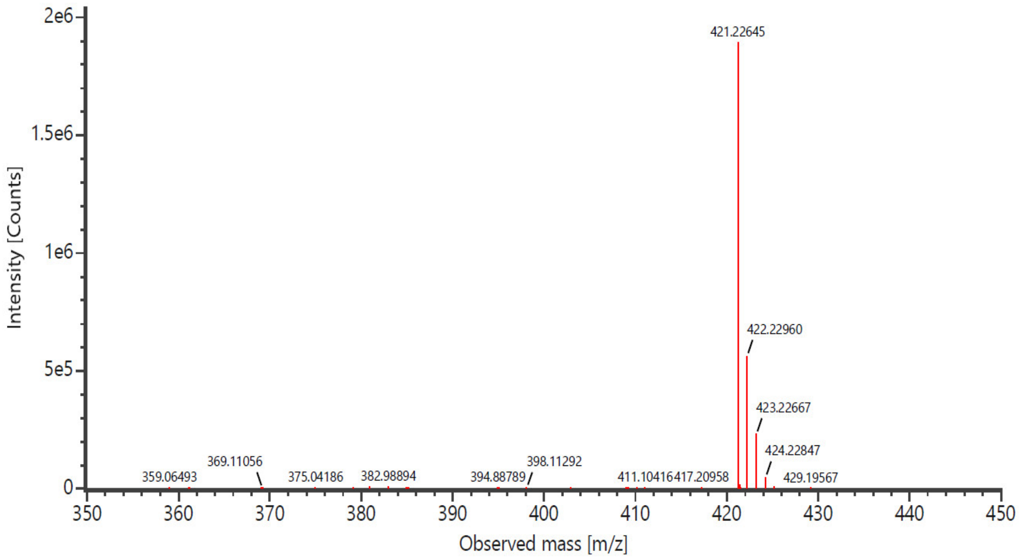

Figure S30: Mass Spectrum (−ESI) of [AoT] in [P_{88814}] [AOT]

3. Characterization of Polymerization

Both tributyl and trioctyl formulation were cured under UV light for 5 hours. The polymerization was confirmed using infrared spectroscopy (IR). The peak of (RH)C=C(H_2) monosubstituted alkene stretch ~910 and ~990 cm^{-1}were missing in each formulation after polymerization.

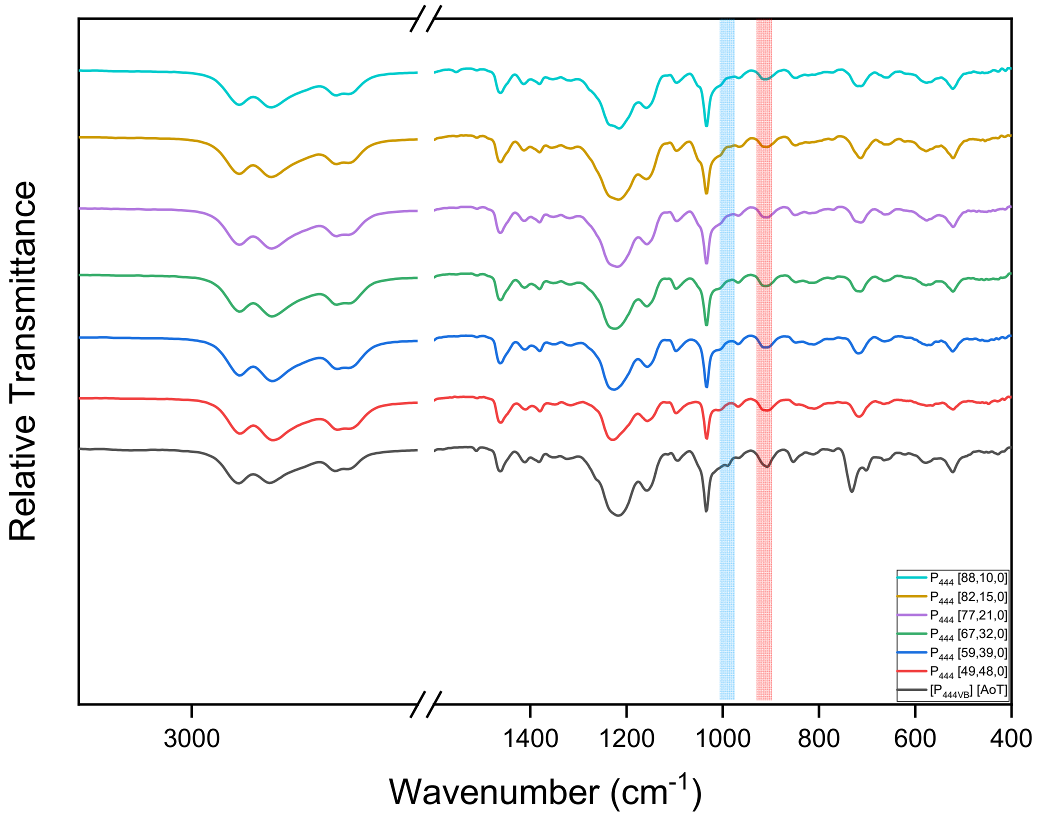

Figure S31: FT-IR comparison of [P_{444VB}][AOT] across samples with various weight percentage of polymerizing ionic liquids (indicated by colors). The absence of (RH)C=C(H_2) stretch around ~910 and ~990 cm^{-1}proves polymerization.

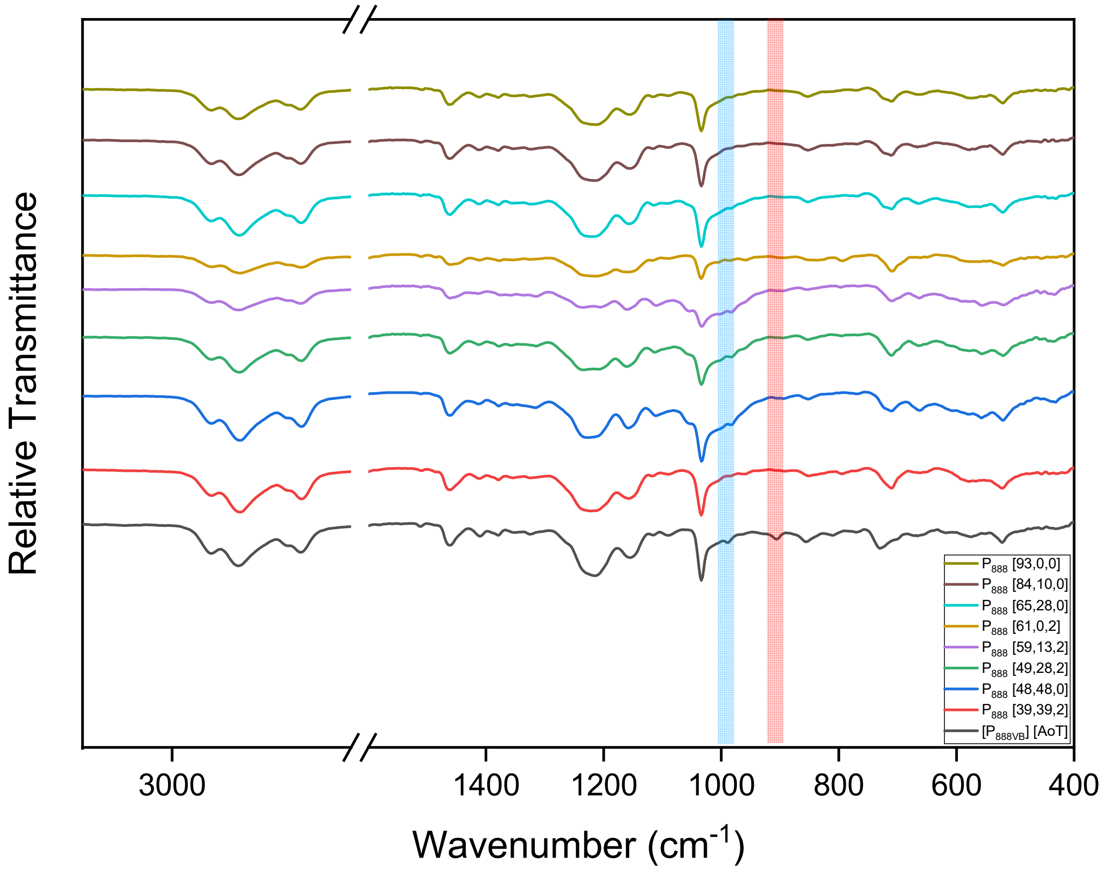

Figure S32: FT-IR comparison of [P_{888VB}][AOT] across samples with various weight percentage of polymerizing ionic liquids (indicated by colors). The absence of (RH)C=C(H_2) stretch around ~910 and ~990 cm^{-1}proves polymerization.

4. Contact Angle Measurement

The evaluation of hydrophobicity involves measuring the water contact angle. The tributyl formulations' contact angle measurement falls between 24 and 52 degrees, showing a substantially lesser hydrophobicity. In contrast to the tributyl samples, the trioctylphosphonium formulations show contact angles ranging from 83° to 121°, showing a higher level of hydrophobicity. The samples move towards the hydrophilic end when biocides are added to the formulation.

Table S9: Advancing water contact angle measurement of tributylphosphonium and trioctylphosphonium formulations. The pictures show the point of contact between water droplet and the surface of both trialkylphosphonium samples.

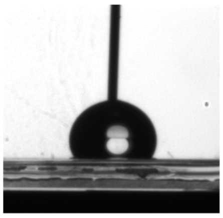 P_{888}[93,0,0]	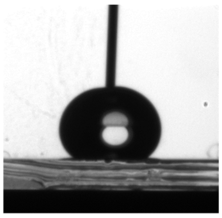 P_{888}[84,10,0]	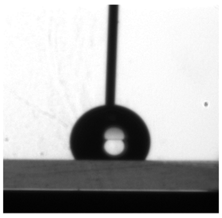 P_{888}[65,28,0]
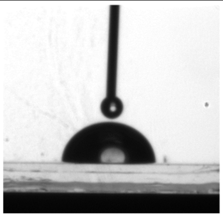 P_{888}[48,48,0]	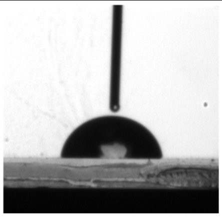 P_{888}[61,0,2]	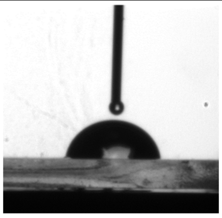 P_{888}[59,13,2]
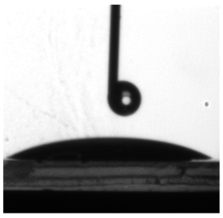 P_{888}[49,28,2]	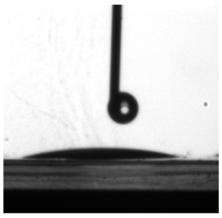 P_{888}[39,39,2]	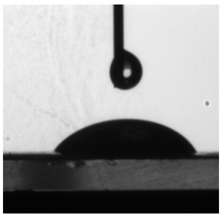 P_{444}[88,10,0]
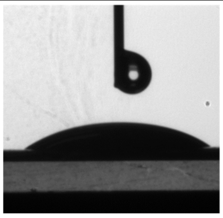 P_{444}[82,15,0]	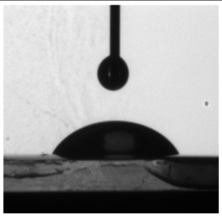 P_{444}[77,21,0]	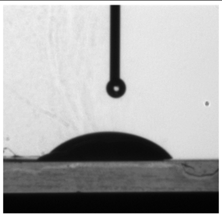 P_{444}[67,32,0]
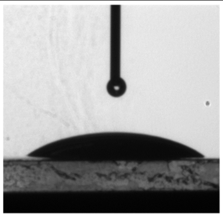 P_{444}[59,39,0]	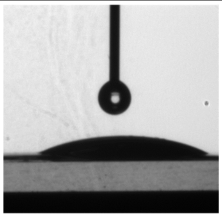 P_{444}[49,48,0]	

5. Micro Testing

The bacterial inhibition of the ionic liquid gels was determined by exposing the trioctylphosphonium gels coatings to both *S. epidermidis* (gram-positive) and *E. coli* (gram-negative) bacteria. Plain filter paper disk (Whatman No.1) was used as the control (no inhibition) for the experiment.

5.1. Fluorescence Assay

Table S10 displays the trioctylphosphine sample fluorescence data. The average mean of five replicated results for each sample, considering both *S. epidermidis* and *E. coli*, is shown in the column headed "Mean Fluorescence." The relative fluorescence of each sample in relation to the experiment's control is shown in the third column. **Table S 11** shows the absorbance value of *S. epidermidis* and *E. coli* after 24h of treatment with trioctylphosphonium samples in LB broth. (*Note: The author didn't perform a blank LB for the absorbance reading measurement after 24h of IL inhibition*). The relative fluorescence proves that the trioctylphosphonium formulation inhibit the growth of *S. epidermidis.*

Table S10: Fluorescence Reading of Trioctylphosphonium formulation samples for both *E. coli* and *S. epidermidis.*

SAMPLE	E. *coli*				S. *epidermidis*			
	Mean	**Std dev**	**Rel. Mean**	**Rel. std dev**	***Mean***	**Std dev**	**Rel. Mean**	**Rel. Std dev**
CONTROL	72308.8	11416.6	1	0.157887	237015	76928.78	1	0.324573
P_{888}[93,0,0]	176058.6	16945.58	2.434816	0.23435	56964.6	10446.32	0.240342	0.044075
P_{888}[84,10,0]	149875.8	30235.57	2.072719	0.418145	106752.8	18442.75	0.450405	0.077813
P_{888}[65,28,0]	167035.6	11962.33	2.310031	0.165434	72852.2	17539.55	0.307374	0.074002
P_{888}[48,48,0]	150828.2	19574.28	2.08589	0.270704	100003.6	20167.35	0.421929	0.085089
P_{888}[61,0,2]	101798.6	18130.63	1.407831	0.250739	165789.6	44577.66	0.69949	0.188079
P_{888}[59,13,2]	136111.8	24408.14	1.882368	0.337554	154730.8	31361.5	0.652831	0.132319
P_{888}[49,28,2]	126453.2	49467.97	1.748794	0.684121	137536.6	36329.89	0.580286	0.153281
P_{888}[39.39,2]	139915	18129.39	1.934965	0.250722	185069	44677.16	0.780832	0.188499

Table S11: Absorbance Reading of Trioctylphosphonium formulation samples for both *E. coli* and *S. epidermidis*.

SAMPLE	E. *coli*				S. *epidermidis*			
	Mean	**Std dev**	**Rel. Mean**	**Rel. std dev**	***Mean***	**Std dev**	**Rel. Mean**	**Rel. Std dev**
CONTROL	0.6024	0.135983	1	0.225736	1.052	0.068938	1	0.06553
P_{888}[93,0,0]	0.9992	0.203196	1.658699	0.337311	1.0224	0.133619	0.971863	0.127014
P_{888}[84,10,0]	1.0986	0.274187	1.823705	0.455158	0.8992	0.420814	0.854753	0.400013
P_{888}[65,28,0]	1.2008	0.249012	1.99336	0.413366	0.7384	0.214955	0.701901	0.204329
P_{888}[48,48,0]	1.0468	0.237989	1.737716	0.395069	0.858	0.230999	0.815589	0.219581
P_{888}[61,0,2]	0.5174	0.189346	0.858898	0.314319	0.4168	0.12904	0.396198	0.122662
P_{888}[59,13,2]	0.6388	0.268975	1.060425	0.446505	0.5442	0.22458	0.5173	0.213479
P_{888}[49,28,2]	0.639	0.137742	1.060757	0.228655	0.4346	0.334597	0.413118	0.318058
P_{888}[39.39,2]	0.6364	0.264828	1.056441	0.439621	0.2402	0.172048	0.228327	0.163544

5.2. Disk Diffusion Assay

The presence of halo around the ionic liquid coating in the agar plate indicate the inhibition capacity of the ionic liquid. **Figure S35** shows trioctylphosphonium samples inhibiting the growth of *S. epidermidis. All samples are performed in triplicates.*

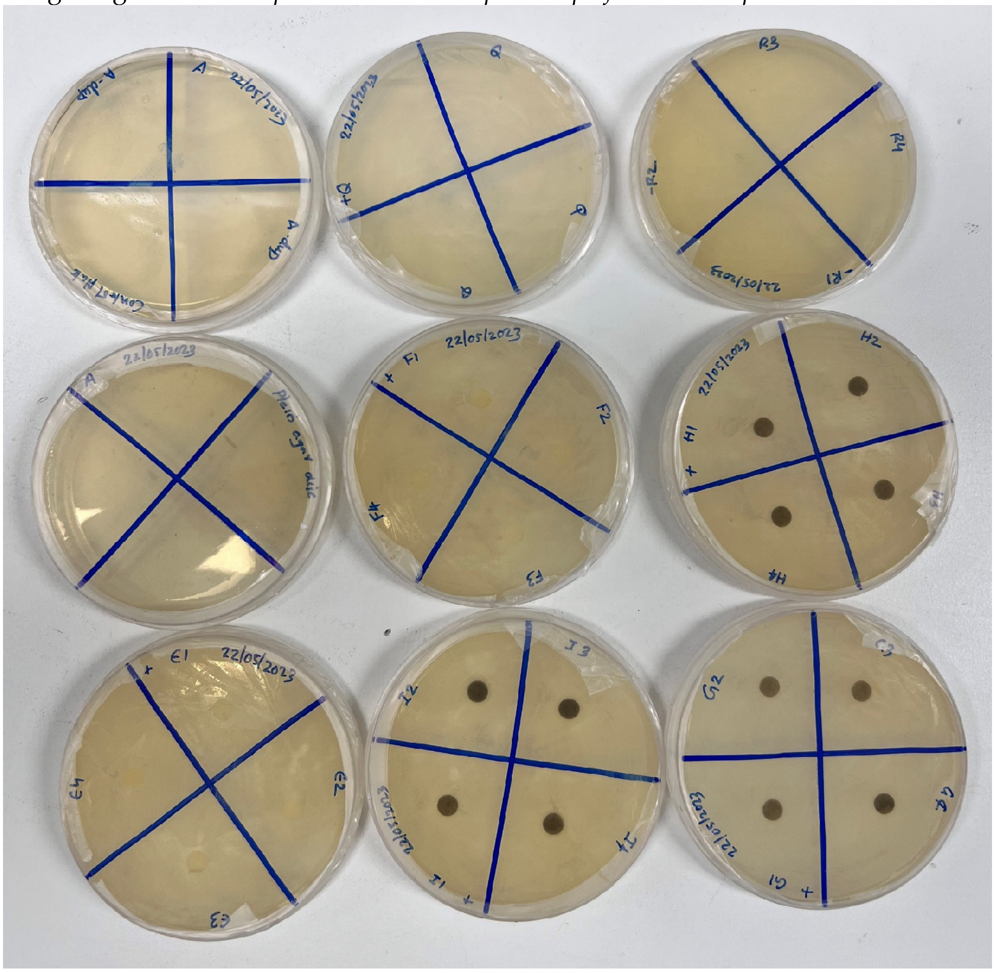

Figure S33: Trioctylphosphonium samples in LB agar plate containing both gram-positive and gram-negative bacteria. A, A dup- plain agar plate (control), Q- LB plate with *S. epidermidis* (control), R- LB plate with *E. coli* (control), E3, E4, F1- P_{888}[93,0,0] in *S. epidermidis*, F2, F3, F4- P_{888}[65,28,0] in *S. epidermidis*, H1, H2- P_{888}[59,13,2] in *S. epidermidis*, H3, H4, I1- P_{888}[49,28,2] in *S. epidermidis*, E1, E2- P_{888}[84,10,0] in *S. epidermidis*, I2, I3, I4- P_{888}[39,39,2] in *S. epidermidis*.

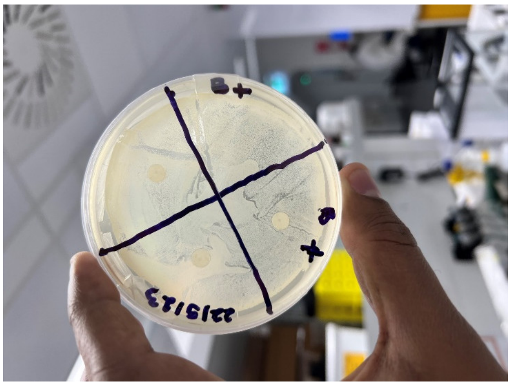

Figure S34: Gram Positive bacteria (S. *epidermidis*) in agar plate with plain disc as control

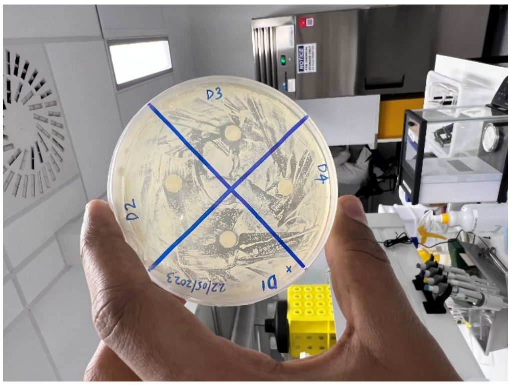

Figure S35: P888[48,48,0] showing inhibition against gram-positive bacteria. The label D1, D2, D3 represents the three replicate of the sample P888[48,48,0].

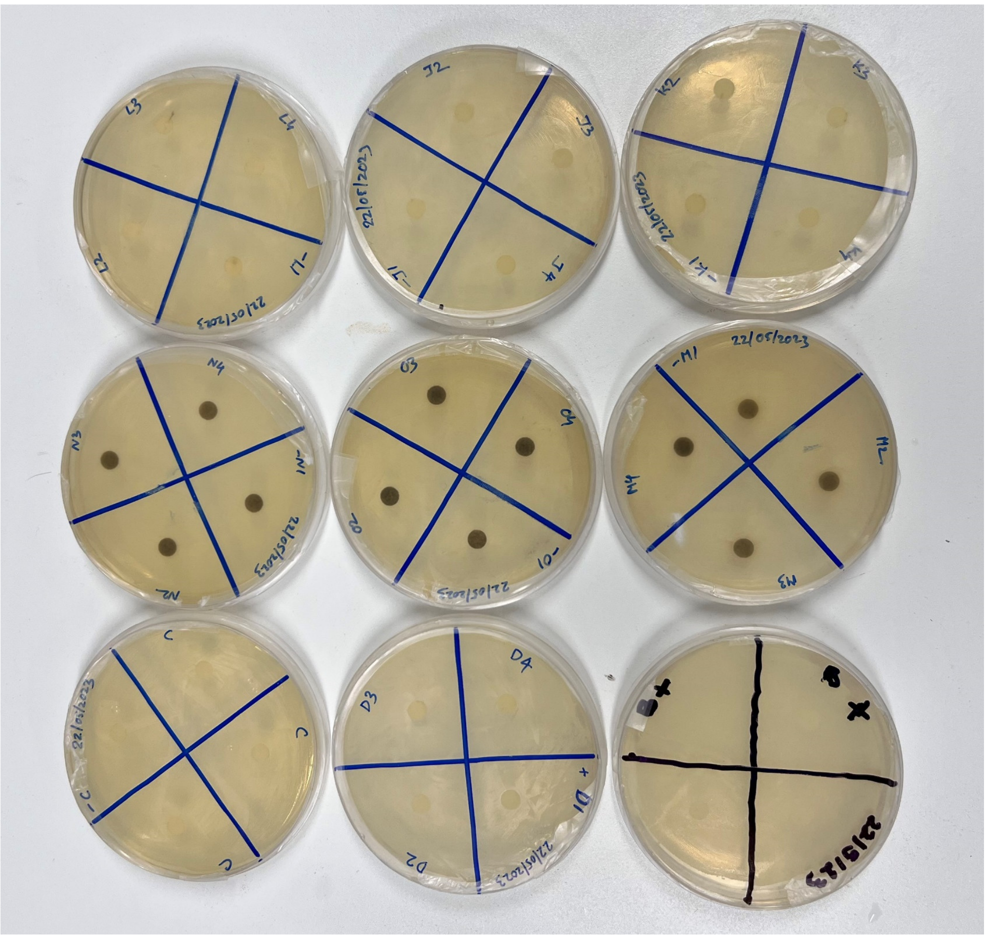

Figure S36: Trioctylphosphonium samples in LB agar plate containing both gram-positive and gram-negative bacteria. B- *S. epidermidis* with plain disk in LB agar, C- *E. coli* with plain disk in LB agar D1, D2, D3- P888[48,48,0] in *S. epidermidis*, D4- P888[84,10,0] in *S. epidermidis*, J1, J2, J3- P888[48,48,0] in *E. coli*, J4, K1, K2- P888[84,10,0] in *E. coli*, K3, K4, L1- P888[93,0,0] in *E. coli*, L2, L3, L4- P888[65,28,0] in *E. coli*, M1, M2, M3- P888[61,0,2] in *E. coli*, M4, N1, N2- P888[59,13,2] in *E. coli*, N3, N4, O1- P888[49,28,2] in *E. coli*, O2, O3, O4- P888[39,39,2] in *E. coli.*

6. Total fouling rate calculation

The total fouling rate is calculated by adding (the percentage of FR) * (the coverage of that FR). The intention behind this metric is to provide an indication of the intensity of fouling. We judge this as useful because it allows comparison of how destructive the fouling is to fuel efficiency and risk of transporting non-native species.

For example, the total foul rating of P_{444}[88,10,0] on day 56 is calculated as follows:

(10x0.15) +(20x0.15) +(30x0.05) +(60x0.10) +(100x0.55) = 67

Table S12: Total fouling rate of tributyl formulations used in the first trial. These coupons were tested in New Zealand coastal waters from December,2022 to February,2023.

Sample	Days in Water	Fouling Rate										Total Fouling Rate (%)
		10	20	30	40	50	60	70	80	90	100	
Epoxy Control	14	2					2					1.4
	28	50		12			12	1				16.5
	56	15	15	5			10				55	67
	92			15							85	89.5
P_{444}[88,10,0]	14	40	60									16
	28	38	15	7		10						13.9
	56	25	30	33				4			8	29.2
	92	45	15	25				7			8	27.9
P_{444}[82,15,0]	14	12										1.2
	28	25	65	2		5						18.6
	56	10	50	33				5				24.4
	92	5	22	65				5			3	30.9
P_{444}[77,21,0]	14	5	20									4.5
	28	50	44	6								15.6
	56	35	8	50	2						5	25.9
	92	2	25	65	3						5	30.9
P_{444}[67,32,0]	14	50			1							5.4
	28	41	40	2			12					19.9
	56	13	26	50				8			2	29.1
	92		8	85							7	34.1
P_{444}[59,39,0]	14	3										0.3
	28	14	80	1		5						20.2
	56	40	22	25				5			4	23.4
	92	5	25	63				5			2	29.9
P_{444}[49,48,0]	14	30	40									11
	28	30	40	1		1						11.8
	56	18	45	33	4							22.3
	92		8	85	3						4	32.3

Table S13: Total fouling rate of tributyl formulation used in the trial. These coupons were tested in New Zealand coastal waters from April, 2023 to May, 2023.

Sample	Days in Water	Fouling Rate										Total fouling Rate (%)
		10	20	30	40	50	60	70	80	90	100	
Epoxy Control	14	30										3
	43	50		25	4							14.1
	63	59		30	8						3	21.1
	84	50		35	10						5	24.5
P_{888}[93,0,0]	14	15										1.5
	43	30	5	7	2							6.9
	63	65	15	12	2							13.9
	84	65	10	15	3	2					3	17.2
P_{888}[84,10,0]	14	15										1.5
	43	25		15	1							7.4
	63	24	50	25	1							20.3
	84	52	10	35	2						1	19.5
P_{888}[65,28,0]	14	20										2
	43	15	3	5	1							4
	63	50	9	6	3							9.8
	84	50	3	6	3	1						8.6
P_{888}[48,48,0]	14	30										3
	43	50	5	7	2							8.9
	63	30	40	15	2							16.3
	84	30	40	10	2						2	16.8
P_{888}[61,0,2]	14	30										3
	43	60		12	2							10.4
	63	60		15	3							11.7
	84	60		18	7							14.2
P_{888}[59,13,2]	14	15										1.5
	43	50		7	1							7.5
	63	50		12	2							9.4
	84	50		25	7							15.3
P_{888}[49,28,2]	14	35										3.5
	43	30	15	3	1							7.3
	63	15	80	3	2							19.2
	84	50	30	5	5							14.5
P_{888}[39,39,2]	14	30		5								4.5
	43	50	5		1							6.4
	63	30	65		1							16.4
	84	65	30	3	2							14.2

6.1. Field Test Picture (Tributyl(vinylbenzyl)phosphonium docusate

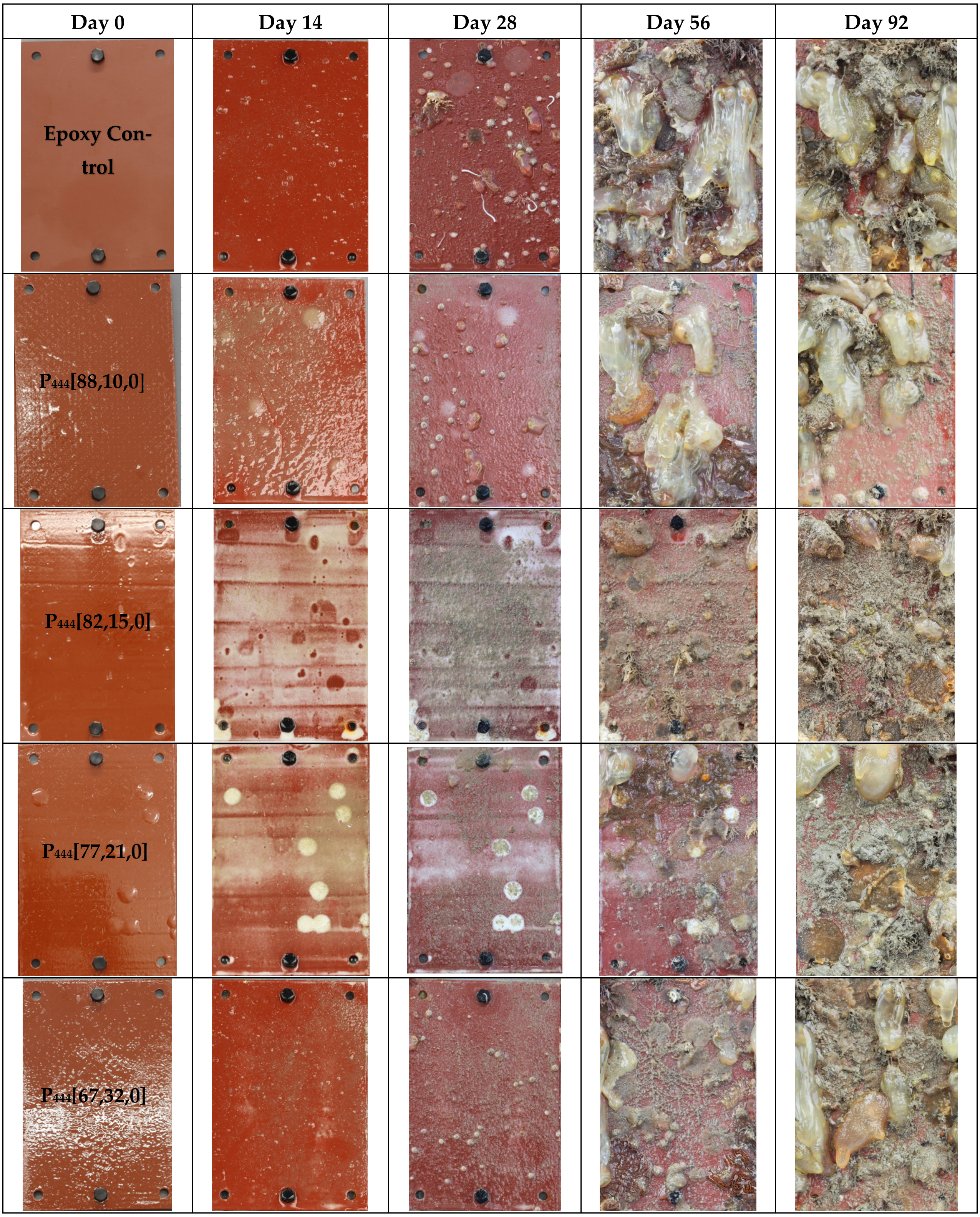
Day 0
Day 14
Day 28
Day 56
Day 92
Epoxy Control
P444[88,10,0]
P444[82,15,0]
P444[77,21,0]
P444[67,32,0]

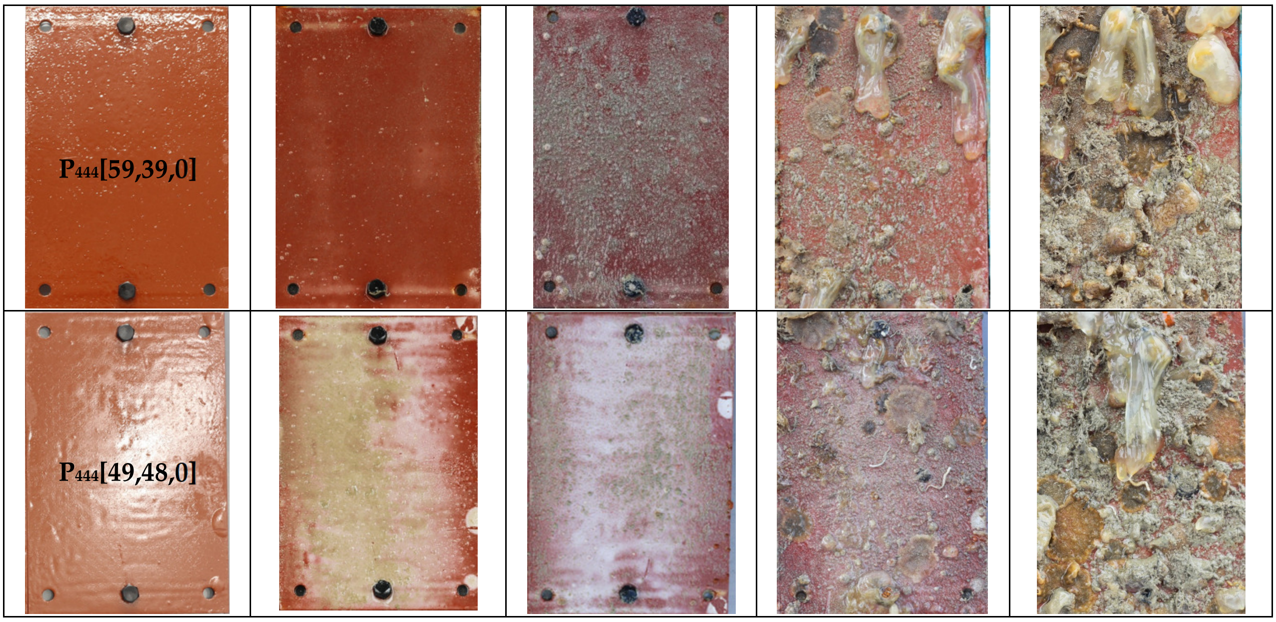

Figure S37: Field trial picture of tributyl(vinylbenzyl)phosphonium docusate formulation along with epoxy treated stainless steel coupon as control on Day 0, Day 14, Day 28, Day 56 and Day 92. These coupons were tested in New Zealand coastal waters from December, 2022 to February, 2023.

7. Surface Roughness of the Gel Samples

The texture of the surface was analysed using a Scanning Electron Microscope (SEM) and 3D Profilometer. The results from both measurements concluded that the Ionic Liquid Gel surface is gel, not a rough surface.

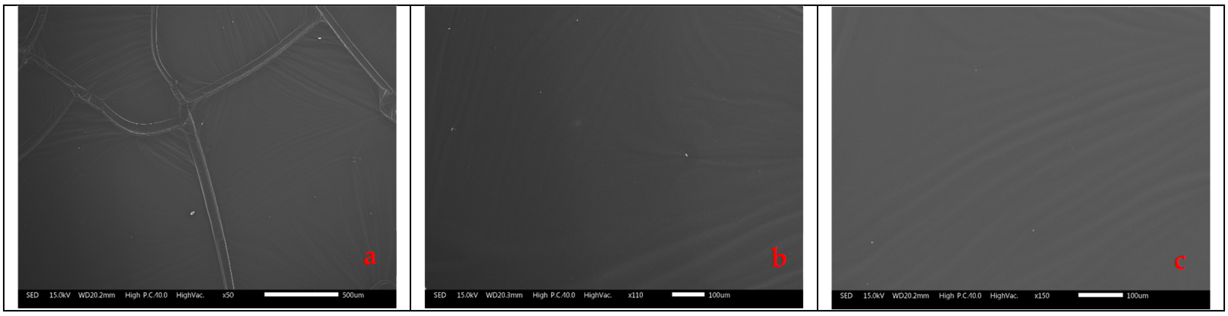

Figure S38: Top view of P_{888}[93,0,0] coated on a glass slide under SEM (a) 50x, (b) 110x, (c) 150x magnification.

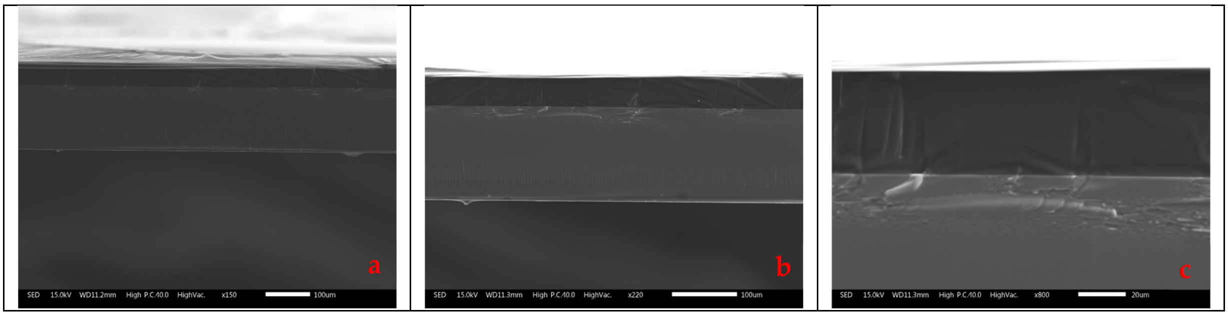

Figure S39: Cross section view of P_{888}[93,0,0] coated on a glass slide under SEM (a) 150x, (b) 220x, (c) 800x magnification.

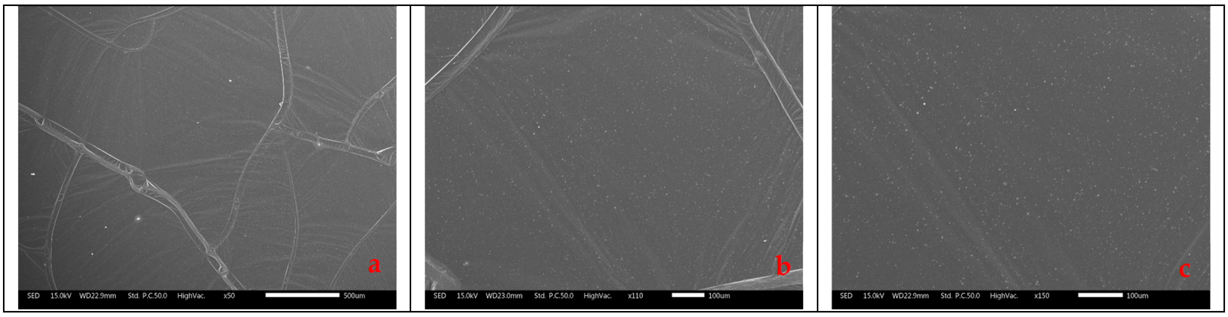

Figure S40: Top view of P_{888}[61,0,2] coated on a glass slide under SEM (a) 50x, (b) 110x, (c) 150x magnification.

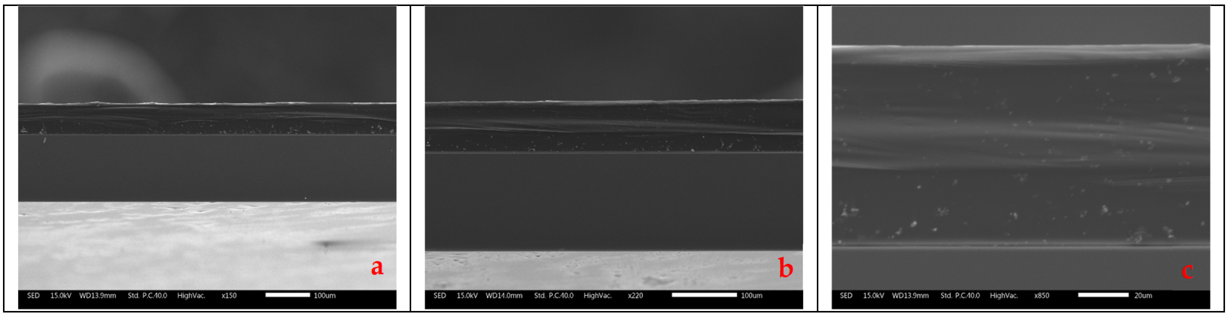

Figure S41: Cross section view of P_{888}[61,0,2] coated on a glass slide under SEM (a) 150x, (b) 220x, (c) 800x magnification.

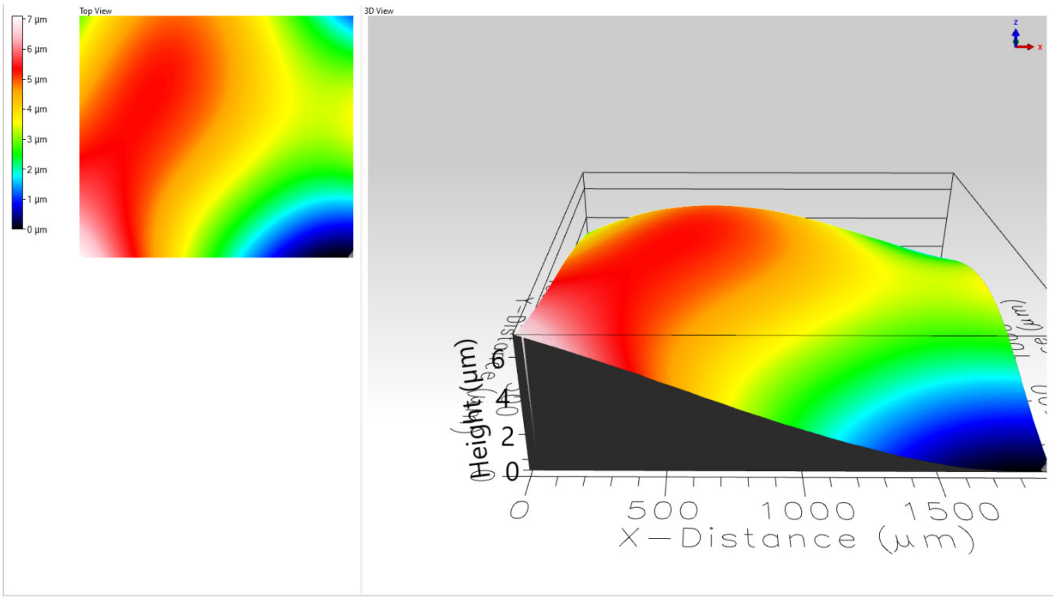

Figure S42: Top view of P_{888}[93,0,0] coated on a glass slide under Profilm3D for measuring roughness.

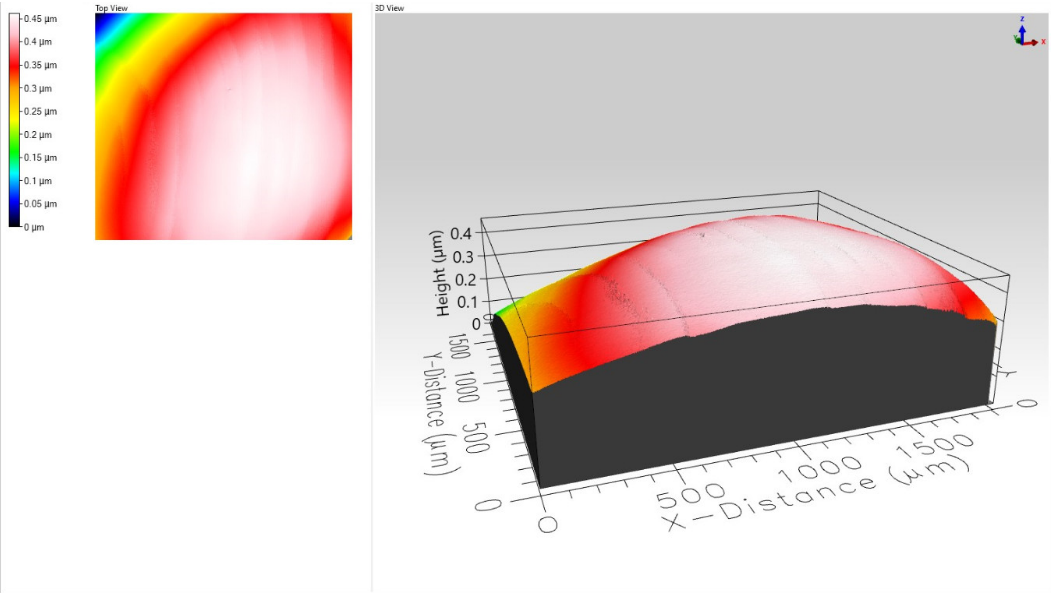

Figure S43: Top view of P_{888}[61,0,2] coated on a glass slide under Profilm3D for roughness measurement.

8. Reference

1. Chemistry: Sulfonates infrared spectra. Available online: http://openchemistryhelp.blogspot.com/2012/12/sulfonates-infrared-spectra.html (accessed on 05 July 2023).
